# Lithium Modulates Autophagy in Esophageal and Colorectal Cancer Cells and Enhances the Efficacy of Therapeutic Agents *In Vitro* and *In Vivo*

**DOI:** 10.1371/journal.pone.0134676

**Published:** 2015-08-06

**Authors:** Tracey R. O’Donovan, Simon Rajendran, Seamus O’Reilly, Gerald C. O’Sullivan, Sharon L. McKenna

**Affiliations:** 1 Leslie C. Quick Laboratory, Cork Cancer Research Centre, BioSciences Institute, University College Cork, Cork, Ireland; 2 Department of Medical Oncology, Cork University Hospital, Cork, Ireland; Swedish Neuroscience Institute, UNITED STATES

## Abstract

Many epithelial cancers, particularly gastrointestinal tract cancers, remain poor prognosis diseases, due to resistance to cytotoxic therapy and local or metastatic recurrence. We have previously shown that apoptosis incompetent esophageal cancer cells induce autophagy in response to chemotherapeutic agents and this can facilitate their recovery. However, known pharmacological inhibitors of autophagy could not enhance cytotoxicity. In this study, we have examined two well known, clinically approved autophagy inducers, rapamycin and lithium, for their effects on chemosensitivity in apoptosis incompetent cancer cells. Both lithium and rapamycin were shown to induce autophagosomes in esophageal and colorectal cancer cells by western blot analysis of LC3 isoforms, morphology and FACS quantitation of Cyto-ID or mCherry-GFP-LC3. Analysis of autophagic flux indicates inefficient autophagosome processing in lithium treated cells, whereas rapamycin treated cells showed efficient flux. Viability and recovery was assessed by clonogenic assays. When combined with the chemotherapeutic agent 5-fluorouracil, rapamycin was protective. In contrast, lithium showed strong enhancement of non-apoptotic cell death. The combination of lithium with 5-fluorouracil or oxaliplatin was then tested in the syngenic mouse (balb/c) colorectal cancer model—CT26. When either chemotherapeutic agent was combined with lithium a significant reduction in tumor volume was achieved. In addition, survival was dramatically increased in the combination group (p < 0.0001), with > 50% of animals achieving long term cure without re-occurrence (> 1 year tumor free). Thus, combination treatment with lithium can substantially improve the efficacy of chemotherapeutic agents in apoptosis deficient cancer cells. Induction of compromised autophagy may contribute to this cytotoxicity.

## Introduction

Macro-autophagy is an evolutionary conserved catabolic process through which a cell recycles its own macromolecules and organelles. Intra-cellular material becomes incorporated into double membraned vesicles termed autophagosomes, which are subsequently trafficked to the lysosome for degradation. Autophagy has now emerged as a key process, which can modulate tumorigenesis and response to therapy. It can be tumor suppressive as it clears damaged organelles and maintains the integrity of a cell under stress. However, when a tumor is established, autophagy has been shown to be an important survival pathway as it protects against a variety of stresses including, hypoxia, metabolic stress, anoikis and chemotherapy [1]. In addition, excessive autophagy has been reported to induce Type II cell death which is a critical cell death pathway and a target for therapeutic intervention, particularly in tumors that have lost apoptosis competency [2] [3]. Autophagy is therefore an adaptive response that exhibits a high degree of plasticity and has context dependent roles in tumor cell biology. It remains unclear how best to modulate it for therapeutic gain.

We have previously demonstrated the importance of autophagy in therapeutic resistance in esophageal cancer. We examined cellular responses in unselected esophageal cancer cell lines treated with chemotherapeutic agents (5-fluorouracil and cisplatin). Drug sensitive cells are apoptosis competent and did not recover following withdrawal of drug. In contrast, more resistant cells failed to induce apoptosis, but exhibited autophagy. Some of these cells underwent a Type II-like death process but many recovered following withdrawal of drug. Knockdown of autophagy regulators confirmed the importance of autophagy for this recovery [4]. This contribution of autophagy to cancer cell survival represents a major challenge in chemotherapy. Given that many primary cancers and probably most recurrent cancers are deficient in apoptosis signaling, we need to examine new strategies to limit autophagic survival and/or induce alternative cell death mechanisms.

As autophagy has been reported to have potentially opposing roles in tumor development, two therapeutic strategies are currently being tested in clinical trials. One strategy is to inhibit the cytoprotective role of autophagy, combined with conventional anti-cancer drugs to sensitize the chemoresistant tumor cells to drugs; the other is to activate autophagic pathways and drive the apoptosis-defective tumor cells to undergo an alternative or ‘autophagic’ cell death [5]. Autophagy inducers in clinical trials include; mTOR inhibitors (including rapamycin analogues; everolimus, teserolimus) and autophagy inhibition is primarily being targeted with the lysosomal inhibitors chloroquine or hydroxychloroquine [1]. A major problem with both strategies is that autophagy pathways are still poorly understood, as is the manner in which specific cancers use them. We have only recently begun to understand the high degree of selectivity in the various types of autophagy and the signaling involved. For many of the current trials, it will be difficult to determine the role of autophagy in the success or failure of the regime, as reliable molecular markers of autophagic responses have not been established. In addition, within the limited repertoire of inhibitors and inducers, there are agents with distinct differences in their mechanisms of action (with most being indirect inhibitors/activators) and data is unlikely to be transferable from one autophagy inhibitor and/or inducer to another. Further studies are therefore required to better understand the actions of autophagy modulators in cancer cells.

In our previous study, we tested autophagy inhibitors (3-MA and Bafilomycin) for chemosentizing effects in esophageal cancer cells. None of these agents were effective as chemosensitizers [4]. Here we investigate the effects of two well known, but mechanistically different autophagy inducers, rapamycin and lithium, on chemosensitivity in cancer cells. Both of these agents (or their analogues) are licenced for clinical use. We show that both agents induce autophagy in cancer cells. Rapamycin does not enhance drug sensitivity, whereas lithium shows strong enhancement of toxicity with *in vitro* and *in vivo* models of gastrointestinal cancers.

## Materials and Methods

### Cell Culture and reagents

Established human esophageal cancer cell lines OE19, OE21 and OE33 were obtained directly from the European Collection of Cell Cultures. KYSE450 cells were from DSMZ (Deutsche Sammlung von Mikroorganismen und Zellkulturen GmbH). Murine colon carcinoma cell line CT26 was obtained directly from American Type Culture Collection (ATCC). OE19, OE21 and OE33 cell lines were maintained in RPMI 1640 medium (Sigma R8758), KYSE450 cells were maintained in 50:50 RPMI 1640:F-12 HAMS medium (Sigma N6658), and CT26 cells were maintained in DMEM medium (Sigma D6429). All cultures were supplemented with 1% penicillin/streptomycin (Gibco Life Technologies 15070–063), 10% (v/v) foetal calf serum (Sigma F7524) at 37°C, 5% CO_2_. All reagents were purchased from Sigma unless otherwise stated. Lithium chloride L9650, lithium carbonate 255823, rapamycin R8781, 5-fluorouracil (5-FU) F6627, chloroquine C66288. Oxaliplatin (Eloxatin 5mg/ml) was from Sanofi Aventis.

### Cyto-ID Autophagy detection

Cells were seeded in triplicate (3.0 x 10^4^ cells/cm^2^) and treated with lithium (10–30 mM) or rapamycin (100–300 nM) alone or in combination with chloroquine (10 μM) for 24 and 48 hours in wells of a 6 well plate. Cells were harvested with trypsin and prepared according to manufacturer’s instructions. The Cyto-ID assay (Enzo Life Sciences ENZ-51031-K200) incorporates a 488 nm-excitable green fluorescent detection reagent that specifically fluoresces in autophagic vesicles. An increase in the number of autophagic vesicles, which stain green is detected as an increase in fluorescence in the FL-1 channel. Cells were incubated in Cyto-ID (1 μl Cyto-ID/1ml cell culture medium without phenol red indicator) for 30 minutes and washed prior to analysis by flow cytometry FACScan.

### Western blotting and antibodies

Total cellular protein extracts were prepared by scraping the cells into modified RIPA buffer (50 mM Tris HCl (pH 7.4), 150 mM NaCl, 0.25% sodium deoxycholate, 1% Igepal, 1 mM EDTA, 1x Pefabloc, 1x protease inhibitor cocktail, 1 mM Na_3_VO_4_, 1 mM NaF). All protein samples were separated on NuPAGE 4–12% Bis-Tris gels and electrophoretically transferred onto PVDF membrane (Invitrogen Life Technologies NP0322 and IB401001). Membranes were incubated with anti-LC3 (polyclonal rabbit antibody–MBL PD014, 1:500 dilution), anti-LAMP 1 (monoclonal mouse antibody–Abcam ab25245, 1:5000 dilution), anti-LAMP 2 (polyclonal rabbit antibody—Abcam ab101325, 1:500 dilution) or anti-cathepsin B (mouse monoclonal antibody–Abcam ab58802, 1:500 dilution) antibodies overnight at 4°C and with anti-β-actin (loading control) (Sigma A5441) for one hour at room temperature. Proteins were visualized using relevant IR-Dye conjugated secondary antibodies (Rockland) on the Odyssey IR imaging system (Li-Cor, Cambridge, United Kingdom).

### Evaluation of morphology

To examine cell morphology, treated cells were cytospun onto glass slides and stained using Pro-Diff (Braidwood Laboratories BAPROD1 –fixed and stained with buffered eosin followed by methyl thionins). Apoptotic cell death is characterised by the presence of two or more of the following morphological features: cell shrinkage, chromatin condensation, DNA degradation and fragmentation into ‘apoptotic bodies’, within an intact plasma membrane. Non-apoptotic cell death was identified by clear loss of cytoplasmic material, pyknosis of the nucleus and an intact nuclear membrane. Cytospin images are representative of at least three independent experiments. Images were captured using a DP70 digital microscope camera and Olympus DP-Soft823 version 3.2 software (Mason Technologies Dublin, Ireland). All images are representative of at least three separate experiments.

### Colony formation assay

The ability of cells to recover from treatments and form colonies as a monolayer was assessed using a colony formation assay. Following treatment, all adherent cells were trypsinized, counted and viability determined. Of those viable cells, 1,500 cells were reseeded into a well of a six-well plate (in triplicate). Cells were allowed to adhere and grow for between 10 to 14 days. To visualise colonies, media was removed, cells were fixed in 96% ethanol for 10 minutes and stained with Prodiff solution C (Braidwood Laboratories E310). Plates were scanned using the Odyssey IR imaging system (Li-Cor, Cambridge, United Kingdom) and colonies quantified. Results are presented as integrated intensity ± SEM from at least three independent experiments. When colony numbers were very low, colonies were manually counted.

### pBABE-puro mCherry-EGFP-LC3B expression

Cells were transfected with 1 μg of the pBABE-puro mCherry-EGFP-LC3B expression plasmid (Addgene 22418) using TurboFect transfection reagent (ThermoScientific R0531) as recommended by the manufacturers. Twenty-four hours post transfection, cells were treated with lithium (20–30 mM) or rapamycin (200–300 nM) alone or in combination with chloroquine (10 μM) for 24 and 48 hours. To assess levels of expression of mCherry-EGFP-LC3, cells were analysed through the PE and 488 2 channels of a BD LSRII flow cytometer (BD Bioscience, Oxford, United Kingdom). Mean fluorescence intensity was measured and data is presented as the mean of three independent experiments.

### Animals and tumor induction

All animal experiments were approved by the Animal Ethics Experimentation Committee (AEEC) of University College Cork and the Department of Health (Ethics number 2010/009), in accordance with guidelines set out by European Communities (Amendment of cruelty to animals act 1876) regulation 2002 & 2005 (licence number B100/4499). Mice were obtained from Harlan Laboratories (Oxfordshire, England). They were maintained in pathogen free conditions, at a constant room temperature (22°C) with a natural day/night light cycle in a conventional animal colony. Standard laboratory food and water were provided *ad libitum*. Before experiments, the mice were afforded an adaptation period of at least 14 days. Female balb/c mice of 6–8 weeks of age, with weights of 18–22 g were used in all experiments. For routine tumor induction, 1 × 10^6^ CT26 (colorectal carcinoma) cells suspended in 200 μl of serum free DMEM were injected subcutaneously into the right flank, after anesthesia (intraperitoneal (IP) administration of 200 μg xylazine and 2 mg ketamine). All mice were euthanized by cervical dislocation under overdose of anesthesia.

### *In vivo* lithium concentration

Lithium concentrations were selected based on previous *in vivo* studies which are focused on neurological studies. In human studies, 0.6–0.8 mmol/L (mEq/L) has been established as a safe therapeutic dose in patients on long term lithium. Adverse reactions may be encountered at levels below 1.5 mEq/L. Mild to moderate adverse reactions may occur from 1.5 to 2.5 mEq/L, and moderate to severe reactions may be seen at levels of 2.0 mEq/L and above [6]. We could achieve chemosensitization *in vitro* at lithium levels equivalent to 1.6 mmol/L–this is at the higher end of the scale for humans. With regard to animal studies, the following range of concentrations have been previously safely employed (60, 80, 100 and 250 mg/kg) [7–10]. In a study that used comparable lithium concentrations as those used in this work, plasma levels of lithium were reported to be 0.25 mM [11]. This is lower than the therapeutic range in humans (0.5–2.0). We opted for 200 mg/kg, following a titration to assess toxicity. As promising effects were achieved in animals at this concentration–without toxicity–we did not further increase this concentration.

### *In vivo* drug delivery

Mice were randomly divided into experimental groups. Mice were treated at a tumor volume of approximately 100 mm^3^ in volume (5–7 mm major diameter). All treatments were delivered in 50ul volumes given either directly into the tumor, or via IP injection every three days. Treatment groups were as follows; PBS (Control), lithium chloride (200 mg/kg), 5-FU (20 mg/kg), oxaliplatin (10 mg/kg) and combinations of either lithium and 5-FU (200 mg/kg, 20 mg/kg) or lithium and oxaliplatin (200 mg/kg, 10 mg/kg). Lithium was reconstituted in sterile phosphate buffered saline. Fluorouracil was supplied by Hospira Ireland (25 mg/ml solution for injection). Oxaliplatin was supplied by Sanofi Ireland (5 mg/ml). All drugs were injected using aseptic techniques to avoid contamination. Following drug treatment, tumors were monitored by alternate day measurements in two dimensions, using vernier callipers. Tumor volume was calculated according to the formula V = ab^2^ᴨ/6, where ‘a’ is the longest diameter of the tumor and ‘b’ is the longest diameter perpendicular to diameter ‘a’. Animals were culled when tumor volumes exceeded ~ 500/600 mm^3^ (no greater than 15 mm in diameter). The toxicity of lithium *in vivo* was tested by examining the plasma levels of sodium, potassium and creatinine in all treatment groups, 24 hours after the final treatment of a three week study. There was no difference in the levels of sodium, potassium or creatinine in any of the treatment groups (PBS control, lithium, 5-FU, 5-FU & lithium). This coupled with the impeccable health of the animals indicates that levels of lithium are below those associated with toxicity. During the experiments, monitoring of animal health (measurement of body weight, eating behavior, drinking behavior, body waste, and the condition of the skin and hair coat) was carried out every day.

### MTT viability assay

Cells were seeded at 3 X 10^4^ cells per cm^2^, treated for 48 hours and incubated for an additional 60 minutes at 37°C in 0.5 mg/ml MTT dye (Sigma M2128). Viable, metabolizing cells reduce MTT dye, producing a dark formazan product, with absorbance read at 562 nm, reference wavelength 620 nm.

### Statistical analysis

Statistical analysis was carried out using GraphPad Prism 5 software. Kaplan-Meier survival curves were used to assess impact of treatment on overall survival. The log-rank (Mantel–Cox) test was used to compare survival distributions of two sample groups. The p-value was considered statistically significant when it was less than 0.05. ** p < 0.01, *** p < 0.0001. Median survival of each sample group was also assessed. Student’s t-test (unpaired) was used to assess significance of mean tumor volume on the final day of treatment. ** p < 0.005.

## Results

### Evaluation of the effects of lithium and rapamycin on autophagy induction in KYSE450 esophageal cells

We previously employed four esophageal cell lines to investigate the consequences of apoptotic and autophagic responses to drug treatment [4]. Two cell lines (OE21 and OE33) were shown to be apoptosis and autophagy competent and are relatively drug sensitive. In contrast, two cell lines (KYSE450 and OE19) that respond with autophagy alone are more drug resistant and can recover following drug withdrawal. The latter two cell lines are of particular interest as they are likely to be more representative of tumors that have lost apoptosis competency. We therefore evaluated the effects of two mechanistically distinct autophagy inducers, initially on the KYSE450 cells.

Rapamycin is a known mTOR inhibitor [12], whereas lithium has been reported to induce autophagy by inhibiting inositol monophosphatase (IMPase) [13]. We verified that both lithium (lithium chloride unless otherwise stated) and rapamycin induced autophagy in KYSE450 cells using the Cyto-ID autophagy detection assay, assessment of LC3 I and II isoforms by western blotting and by morphological assessment of vesicle accumulation.

**Fig 1:**
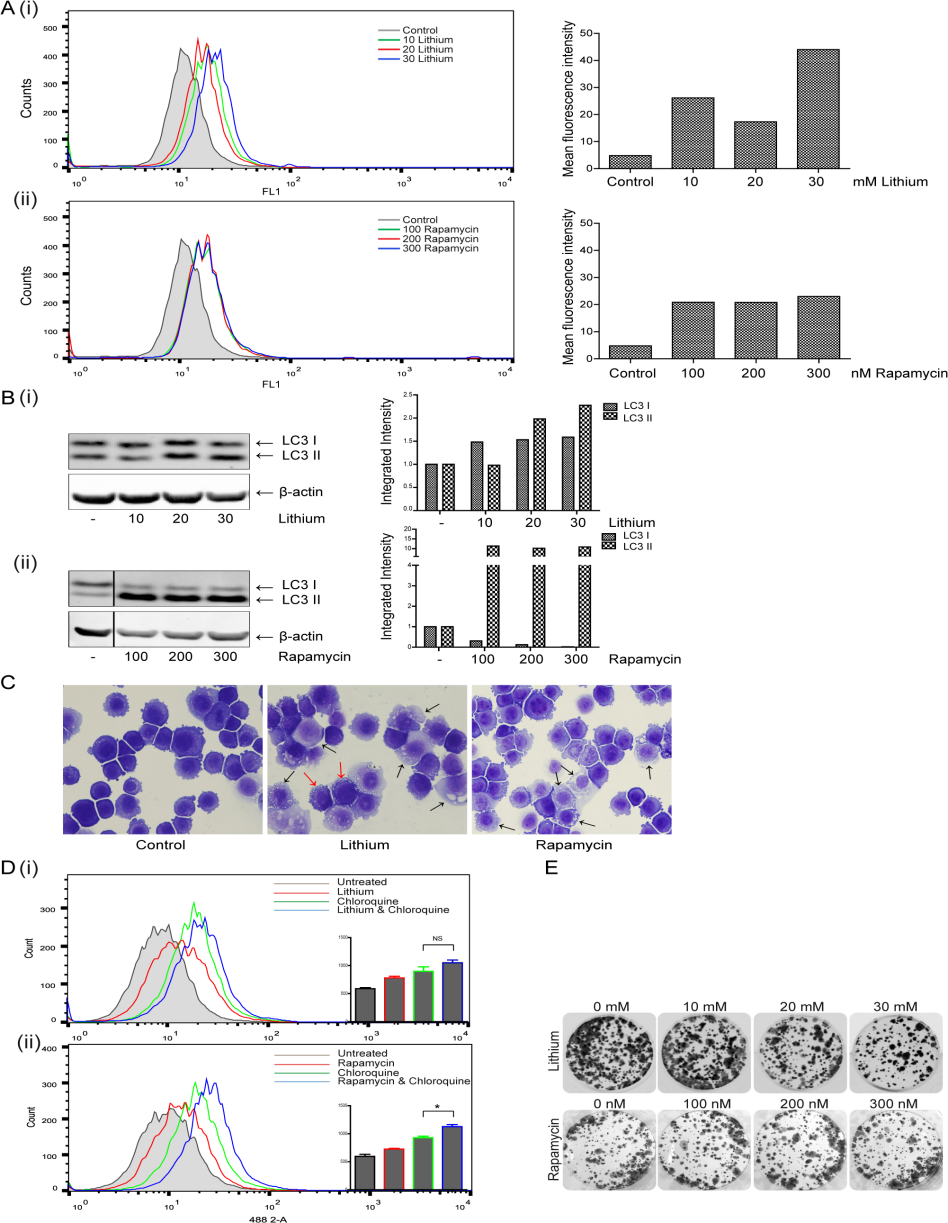
Evaluation of the effects of Lithium and Rapamycin on autophagy induction in esophageal cancer cells. (A) KYSE450 cells were treated with lithium chloride (lithium) (10–30 mM) or rapamycin (100–300 nM) for 24 and 48 hours. Cells were assessed for autophagy induction 24 hours after treatment with lithium (i) or rapamycin (ii) with the Cyto-ID autophagy detection kit. Panels on the left show representative images of FACS analysis, with panels to the right showing corresponding mean fluorescence intensity. Data shown here is representative of three independent experiments. (B) Western blot analysis of LC3 expression in cells treated with lithium (i) or rapamycin (ii) for 24 hours. LC3I and LC3II bands were quantified using the Odyssey Infrared Imaging System (Li-COR), normalized to β-actin and presented as integrated intensities. (C) Morphological features of KYSE450 cells following lithium (30 mM, center panel) or rapamycin (300 nM, right hand panel) treatment for 24 hours. Black arrows indicate accumulation of vesicles in both lithium and rapamycin treated cells, red arrows denote peripheral vesicle accumulation (Magnification 40x). (D) Cells were pretreated with chloroquine (10 μM) for two hours, prior to treatment with lithium (10 mM) (i) or rapamycin (100 nM) (ii) for 48 hours and autophagy levels assessed with the Cyto-ID autophagy detection kit. Representative images of FACS analysis are shown (n = 3), with inset bar graphs showing corresponding mean fluorescence intensity (* p < 0.05). E Viable cells following treatment with either lithium or rapamycin for 48 hours were counted and equal numbers (1,500 cells per well) reseeded in triplicate, in the absence of drug. Cells were allowed to grow for 14 days, then were fixed and stained and colony regrowth assessed.

Increased Cyto-ID fluorescence indicated autophagosome formation in lithium (10, 20 and 30 mM) and rapamycin (100, 200 and 300 nM) treated cells, at 24 hours [Fig 1A (i) and (ii)] respectively. Lithium induced autophagy was more pronounced than that induced by rapamycin with a 9.2-fold increase observed between control and 30 mM lithium treated cells, compared to the 4.8-fold increase induced by the higher concentration of rapamycin. The data shown is representative of three independent experiments. After 48 hours of treatment, Cyto-ID detectable autophagy was reduced in lithium treated cells (2.3-fold increase observed between control and 30 mM treated cells) and undetectable in the rapamycin treated cells (**data not shown**).

Western blot analysis confirmed an increase in the levels of both LC3 I and II following 24 hour treatment with lithium [Fig 1B (i)], an effect that was maintained for up to 48 hours (data not shown). The levels of LC3 II were also significantly raised by rapamycin at 24 hours [Fig 1B (ii)], and were partially reduced 48 hours post treatment (data not shown).

Morphological analysis of KYSE450 cells confirmed accumulation of vesicles in the cytoplasm of cells treated with lithium (30 mM) (Fig 1C, middle panel) and rapamycin (300 nM) (Fig 1C, right panel) for 48 hours (also obvious at 24 hours, data not shown). Arrows indicate cells with significant vesicle accumulation. Lithium treated cells exhibited extensive peripheral vesicle accumulation (red arrows).

Autophagic flux is the term used to describe the entire process of autophagy, including delivery of sequestered components to the lysosome and their subsequent breakdown [14]. If the accumulation of autophagosomes is due solely to a failure of cells to turnover autophagosomes, the addition of chloroquine–a lysosomotropic agent which inhibits lysosomal activity and blocks autophagosome turnover [15], will have no further effect on vesicle accumulation. Therefore, to differentiate between autophagy induction and vesicle accumulation due to a failure in turnover, we pre-treated the cells with chloroquine (10 μM) prior to treatment with either lithium (10 mM) or rapamycin (100 nM) for 48 hours. As shown in Fig 1D, treatment of KYSE450 cells with chloroquine alone, as expected, caused an increase in the accumulation of vesicles (green overlays) as assessed by Cyto-ID. Lithium alone, caused a significant increase in vesicle accumulation, which was enhanced, although not significantly, by chloroquine (blue overlay), suggesting only moderate flux. In contrast, rapamycin alone caused a more modest shift in detectable autophagosomes. The addition of chloroquine, significantly increased the level of vesicles (* p = 0.0136) (blue overlay), indicating autophagosome induction and turnover (flux) in response to rapamycin.

Importantly, the induction of autophagy for up to 48 hours in response to lithium or rapamycin alone, at a range of concentrations, had a limited effect on viability as assessed by colony formation assay (Fig 1E). Some toxicity with lithium is evident above 20 mM. Collectively, these data indicate that both compounds can induce relatively non-toxic accumulation of autophagosomes in these esophageal cancer cells.

### Assessment of the effect of rapamycin and lithium on chemosensitivity

We then evaluated the effects of combining rapamycin or lithium with 5-FU on sensitivity of esophageal cell lines. Autophagy induction by 5-FU in the KYSE450 cells was previously demonstrated by GFP-LC3, electron microscopy, and western blot analysis of LC3II [4]. Autophagic flux, induced by 5-fluorouracil (5-FU) has also been included here, showing an enhancement of vesicle accumulation with chloroquine (S1 Fig blue overlay).

**Fig 2:**
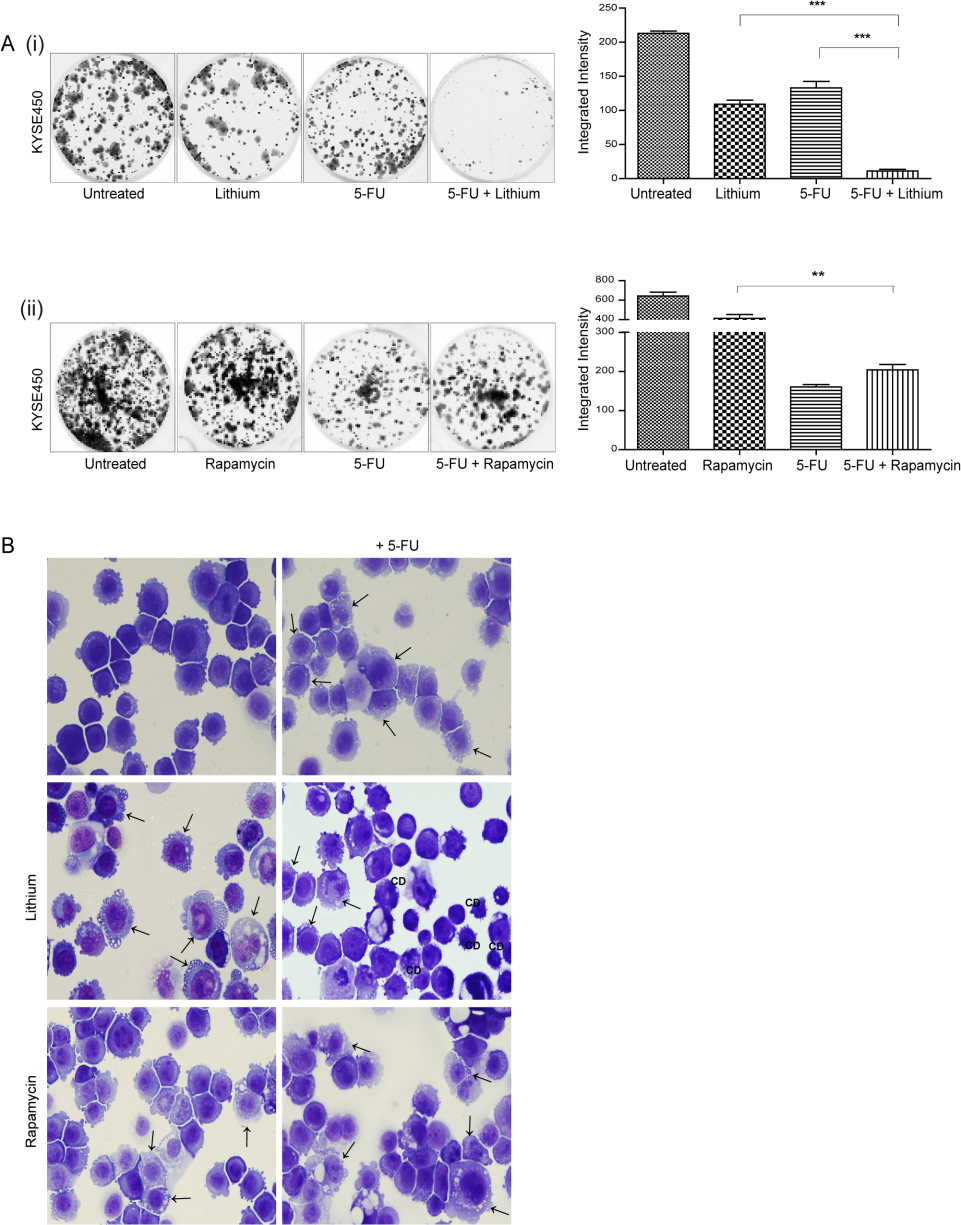
Assessment of the effect of autophagy inducers on chemosensitivity in human esophageal cancer cells. KYSE450 cells were treated with lithium chloride (lithium) (30 mM) or rapamycin (300 nM) alone or in combination with 5-fluorouracil (5-FU) (40 μM) for 48 hours. (A**)** Viable cells following treatment with either lithium (i) or rapamycin (ii) alone or in combination with 5-FU for 48 hours were counted and equal numbers reseeded in triplicate, in the absence of drug. Cells were allowed to grow for 14 days, then were fixed and stained and colonies quantified using the Odyssey Infrared Imaging System (Li-COR). Data is presented graphically as the mean +/- SEM of three independent experiments. *Asterisks* indicate a significant difference in the number of colonies formed in combination treated cells compared to single agent treated cells (*** p < 0.0005, ** p < 0.005) (unpaired *t*-test). (B) Morphological features of KYSE450 cells were examined following treatment for 48 hours. Panels to the left show the morphology induced in response to lithium or rapamycin alone, panels to the right show morphology with the addition of 5-FU. Arrows indicate the accumulation of autophagic vesicles in lithium, rapamycin, 5-FU and combination treated cells. CD denotes the presence of a non-apoptotic cell death morphology (Magnification 40x).

KYSE450 cells were treated with 30 mM lithium chloride, 300 nM rapamycin, 40 μM 5-FU or a combination of autophagy inducer and 5-FU for up to 48 hours. Equal numbers of viable cells from each treatment group were seeded following drug treatment and incubated for up to two weeks, in the absence of drug. The colonies that developed are shown in Fig 2A. Colonies were rare in KYSE450 cells treated with the combination of 5-FU and lithium, with an 11.9-fold decrease in the number of colonies recovering from lithium & 5-FU treatment compared to 5-FU alone (*** p = 0.001) [Fig 2A (i)]. In contrast, the addition of rapamycin to 5-FU treated cells enhanced their ability to recover, with a 2.3-fold increase in the number of returning colonies in rapamycin & 5-FU treated cells compared to 5-FU alone [Fig 2A (ii)].

Morphological analysis revealed that while lithium alone caused an accumulation of cytoplasmic vesicles (left centre panel, arrows), there was a significant increase in the number of cells displaying features of non-apoptotic cell death (CD) in lithium & 5-FU treated cells (right centre panel, CD). Rapamycin alone or in combination with 5-FU, caused an accumulation of cytoplasmic vesicles (arrows), which was not associated with the development of cell death morphologies (Fig 2B lower panels).

To verify that this effect was neither cell line nor 5-FU specific, we confirmed the chemosensitizing effect of lithium in an additional drug resistant/apoptosis incompetent esophageal cancer cell line (OE19) and in combination with an additional chemotherapeutic drug–cisplatin (S2 Fig), which we had previously confirmed to be an inducer of autophagy in these cells [4].

Collectively, these findings highlight that although both lithium and rapamycin can modulate autophagy, they have opposing activity when combined with a chemotherapeutic agent; lithium acts as a powerful chemosensitizer while rapamycin is protective.

We also assessed the effects of combination treatments in apoptosis competent esophageal cell lines. It is important that these agents would not interfere with apoptosis and reduce drug sensitivity. The combination of lithium or rapamycin with 5-FU enhanced autophagy and apoptosis in both OE21 (S3 Fig) and OE33 (data not shown) cells, which was clearly evident by morphology [S3A (i) & S3B (i) Fig]. Both combination treatments also reduced viability as determined by MTT assay [S3A (ii) & S3B (ii) Fig].

A number of lithium salts are used as mood-stabilizing drugs, with lithium carbonate most commonly prescribed due to lower toxicity. To verify that the effects of lithium on autophagy and chemosensitization were not limited to lithium chloride, we treated KYSE450 cells with a range of lithium carbonate concentrations (10–20 mM). Data presented in S4 Fig demonstrates that lithium carbonate induced increased expression of LC3 II on western blot (S4A Fig lanes 2,3 & 4). Immunofluorescence staining for LC3 (S4B Fig centre panel) also indicated a striking accumulation of cytoplasmic vesicles at concentrations as low as 10 mM, with a distribution pattern similar to that observed in lithium chloride treated cells (S4C Fig right panel). When combined with 5-FU, lithium carbonate induced the same cell death morphologies (CD, lower right panels) as those observed in lithium chloride treated cells. Importantly, lithium carbonate also induces similar chemosensitization with 5-FU (40 μM) in KYSE450 cells, notably at the lower concentrations of 10 and 15 mM (S4D Fig). Collectively these findings underscore the important role of the lithium ion as an autophagy modulator and its association with enhancement of cell death in the combination treatment.

### Mechanism of cytotoxicity

Previous studies have suggested that alterations in autophagic flux, leading to an excessive accumulation of autophagosomes can turn autophagy into a destructive process [16]. Our initial studies with the Cyto-ID assay demonstrated that lithium affected autophagosome turnover (Fig 1). We therefore looked for further evidence of downstream differences in autophagosome processing that may account for the ability of lithium to chemosensitize esophageal cells.

We compared the level of flux in lithium and rapamycin treated cells using the fusion protein (mCherry-GFP-LC3) in KYSE450 cells, quantifying both green and red fluorescence by flow cytometry, with two separate lasers to eliminate any compensation requirements. Green and red fluorescence are detected in autophagosomes whereas only red fluorescence is found in autolysosomes due to the pH sensitivity of GFP.

**Fig 3:**
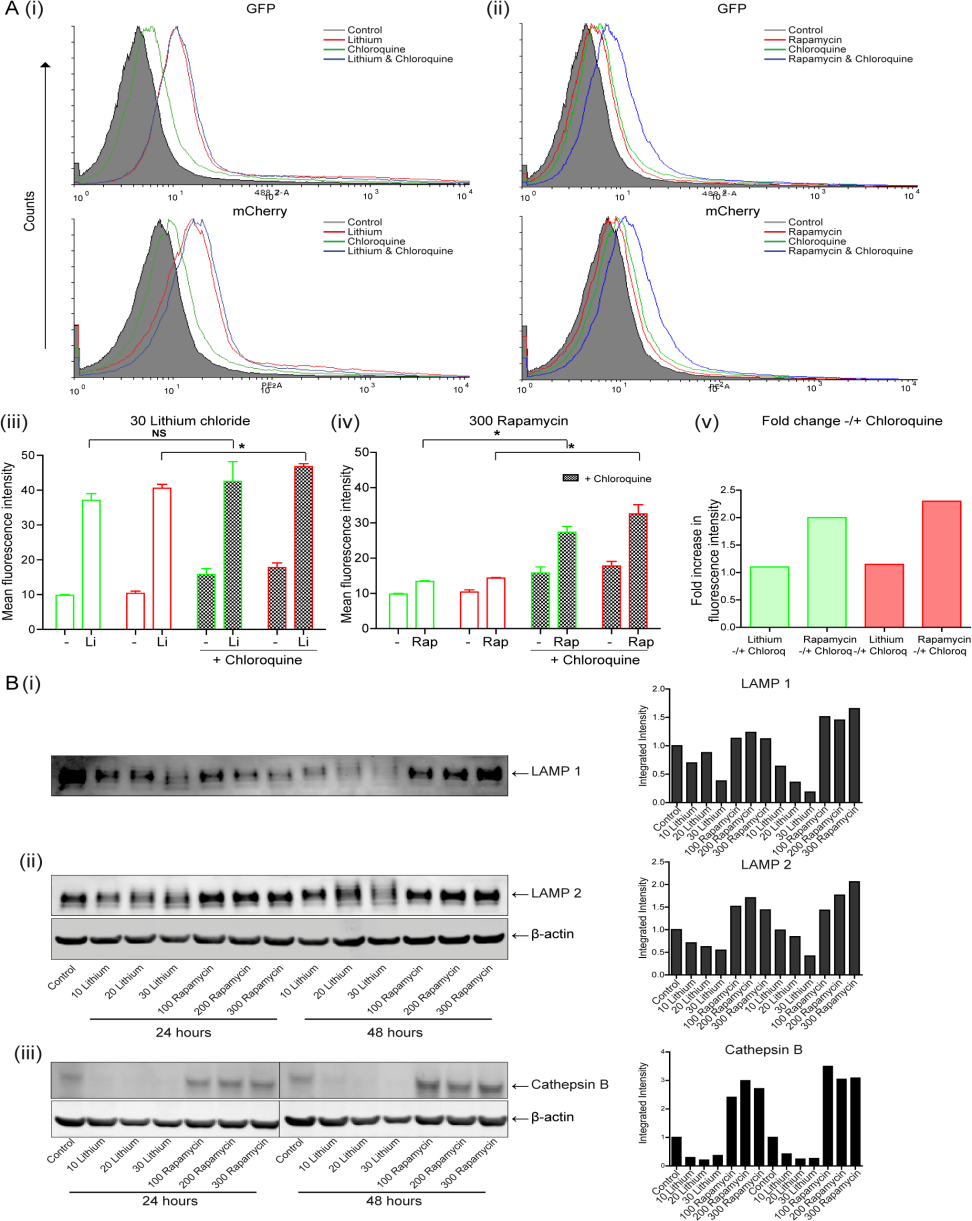
Examination of the autophagosome processing in esophageal cells following treatment with lithium or rapamycin. (A) KYSE450 cells were transfected with pBABE-puro mCherry-EGFP-LC3B expression plasmid. Twenty four hours later, cells were treated with lithium (30 mM) or rapamycin (300 nM) alone or in the presence of chloroquine (10 μM) for 48 hours. Following treatment, cells were harvested and analysed by flow cytometry for EGFP (488 2-A) and mCherry (PE-A) expression. Representative histograms of GFP (upper) and mCherry (lower) for both lithium (i) and rapamycin (ii) treated cells, without (red overlays) and with chloroquine (blue overlays) are shown. Data is presented as mean fluorescence intensity (MFI) of three independent experiments for both GFP and mCherry in lithium (iii) and rapamycin (iv) treated cells (n = 3). The fold change in GFP (green bars) and mCherry (red bars) MFIs between lithium or rapamycin, without and with chloroquine is presented to the right **(v)**. *Asterisks* indicate a significant difference in the MFI (* p < 0.05). (B) Western blot analysis of LAMP 1 (i), LAMP 2 (ii) and cathepsin B (iii) expression in cells treated with lithium (10–30 mM) or rapamycin (100–300 nM) for 24 and 48 hours. All bands were quantified using the Odyssey Infrared Imaging System (Li-COR), normalized to β-actin and presented as integrated intensities.

Representative histograms of GFP (488 2) and mCherry (PE) fluorescence in lithium (30 mM) and rapamycin (300 nM) treated cells are shown in Fig 3A (i) and (ii) respectively. These data are presented graphically in Fig 3A (iii)–(v). Both lithium and rapamycin cause an accumulation of GFP and mCherry above control untreated transfected cells, indicative of enhanced formation of autophagosomes [Fig 3A (i) (ii) (iii) & (iv)]. The level of autophagosomes induced is greater in lithium (Li) treated cells compared to rapamycin (higher levels of both green and red fluorescence, depicted with red histogram overlays and unfilled green and red bars).

The lysosomotropic agent chloroquine, was added to treated cells to block autophagosome turnover [15]. It has previously been shown that in cells containing mCherry-GFP-LC3, inhibition of the turnover/flux of autophagosomes leads to an elevation of green fluorescence (due to loss of pH-mediated quenching of GFP and degradation in lysosomes) and elevation of Cherry fluorescence due to lack of protein degradation [17]. Treatment of KYSE450 cells with chloroquine (10 μM) alone, as expected, caused a modest increase (1.6- and 1.7-fold) in the mean fluorescence intensity (MFI) of both GFP and mCherry respectively (green overlays and filled bars). We then examined fluorescence in lithium and rapamycin treated cells in the presence of chloroquine. The fold change in MFI is plotted in Fig 3A (v). The addition of chloroquine (blue overlays) caused only a modest increase of 1.1- and 1.15-fold in MFI in lithium treated cells in both GFP and mCherry respectively [Fig 3A (iii) & (v)]. This is again suggestive of compromised autophagic flux.

In contrast, the fold increase in MFI in rapamycin treated cells, due to the addition of chloroquine is much greater (blue overlays); with a twofold increase in GFP and a 2.3-fold increase in mCherry [Fig 3A (iv) & (v)]. These data suggest that the autophagosomes produced in rapamycin treated cells are being efficiently turned over by lysosomes, as a block in the pathway enhances accumulation.

It is not clear why chloroquine has minimal effect on lithium treated cells. It is possible that the lysosomes are saturated or defective as the fluorescence is very high in lithium treated cells (NB higher than chloroquine alone).

Other groups using a GFP-LC3 or mCherry-GFP-LC3 fusion protein have described a decrease in GFP when measuring autophagy by flow cytometry, but they also show a decrease in the levels of LC3 on western blot or by immunofluorescence [18, 19]. Our data however, is consistent with the increase in LC3I/II expression observed on western blots with either lithium or rapamycin (Fig 1). A number of other studies corroborate our findings; some report increased expression of GFP-LC3 by FACS [20, 21], while others describe increased expression of both red/green fluorescence measured by FACS and quantitative confocal microscopy with induced autophagy [21] [22–25].

Collectively these data with the mCherry-GFP-LC3 fusion protein, along with the Cyto-ID data shown in Fig 1, suggest induction of autophagy in lithium treated cells, with evidence of saturated or compromised flux.

Other studies in cardiomyocytes have linked impaired autophagic flux with cytotoxicity. These studies showed lysosome deficiencies and down-regulation of LAMP 1 or 2 proteins [26, 27]. Loss of LAMP1, an abundant lysosomal membrane protein is often associated with lysosome consumption. Lamp 2A & B are important for chaperone mediated autophagy and autophagosome—lysosome fusion respectively [28]. We evaluated both LAMP 1 and 2 expression following lithium and rapamycin treatment (Fig 3B). KYSE450 cells were treated with 10, 20, 30 mM lithium and 100, 200, 300 nM rapamycin for 24 and 48 hours. LAMP 1 [Fig 3B (i)] and LAMP 2 [Fig 3B (ii)] expression was evaluated by western blotting. In rapamycin treated cells, both LAMP 1 and 2 expression is similar to untreated cells or elevated–consistent with up-regulation of lysosome biogenesis through mTOR/TFEB signalling [29]. In contrast, lithium treated cells show dramatic depletion of both LAMP 1 and 2, at 24 and 48 hours. We also assessed levels of cathepsin B in lithium and rapamycin treated cells. Rapamycin treated cells show similar or elevated levels of mature/active form of cathepsin B (31 kDA) to the control cells, whereas lithium treated cells show significant depletion of this form of the protein [Fig 3 (iii)]. Deficiency in these key lysosomal proteins is indicative of compromised lysosomal integrity [30, 31].

If compromised flux was solely responsible for the increased cytotoxicity observed it might be expected that a blockage in flux, combined with an autophagy inducer would be cytotoxic. However, we previously demonstrated that the lysosomal inhibitor, bafilomycin could not enhance toxicity with 5-flurouracil (shown to induce autophagy) [4]. We further evaluated whether impeding flux in rapamycin treated cells could enhance cytotoxicity. KYSE450 cells were treated with rapamycin (200 & 300 nM), bafilomycin (1 nM) and 5-FU (30 μM) for 24 and 48 hours. Effects on flux were assessed by western blot analysis of LC3 II and recovery was assessed by clonogenic assay [S5A Fig (i) & (ii)]. No enhancement of cytotoxicity was observed. We also assessed whether the addition of chloroquine (10 μM) to rapamycin treated cells affected viability. Effects on flux were demonstrated by Cyto-ID analysis and recovery was assessed by clonogenic assay [S5B Fig (i) & (ii)]. These data suggest that an impediment in flux is not necessarily a cytotoxic or chemosensitizing event and that other mechanisms are likely to contribute to cytotoxicity in lithium treated cells.

### Evaluation of the effects of 5-fluorouracil (5-FU) and lithium on autophagy induction in murine CT26 colorectal cells

Due to the potential therapeutic benefit of combining lithium with chemotherapy, we decided to assess this combination therapy in a mouse model. As both the immune system and tumor-stromal interactions have been reported to be influenced by autophagy, we selected a syngeneic mouse model to give a more representative reflection of the potential clinical effects of this combination treatment. To date, there are no esophageal derived murine cell lines, so we selected another gastrointestinal (GI) model–CT26 (colorectal) cells grown in a balb/c mouse.

**Fig 4:**
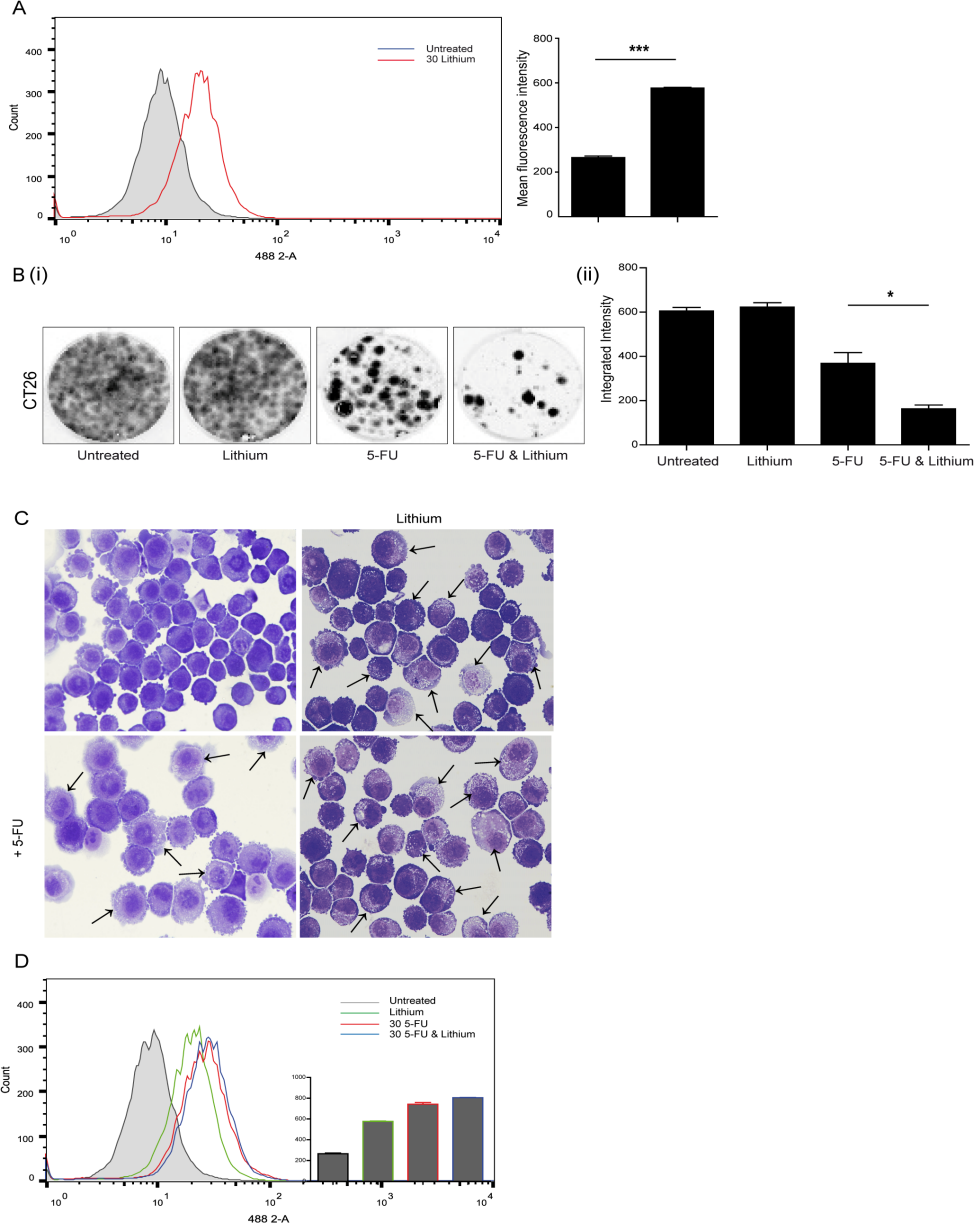
Assessment of the effect of combining autophagy inducers on chemosensitivity in CT26 murine colorectal cells. (A) The induction of autophagy in CT26 cells, following lithium treatment (30 mM) for 24 hours, was assessed with the Cyto-ID autophagy detection kit. (B) Viable cells following treatment with lithium alone or in combination with 5-fluorouracil (5-FU) (20 μM) for 24 hours were counted and equal numbers reseeded in triplicate, in the absence of drug. (i) Cells were allowed to grow for 14 days, then were fixed and stained and colonies quantified using the Odyssey Infrared Imaging System (Li-COR). (ii) Data is presented graphically as the mean +/- SEM of three independent experiments. *Asterisks* indicate a significant difference in the number of colonies formed in combination treated cells compared to single agent treated cells (* p < 0.05) (unpaired *t*-test). (C) Morphological features of CT26 cells were examined following treatment for 24 hours. Panels to the right show the morphology induced in response to lithium alone (upper) and in combination with 5-FU (lower). Lower left panel shows morphology in cells treated with 5-FU alone. Arrows indicate the accumulation of vesicles in lithium, 5-FU and combination treated cells (Magnification 40x). (D) Cells were treated with either lithium (30 mM), 5-FU (20 μM) or a combination of both for 24 hours and autophagy levels assessed with the Cyto-ID autophagy detection kit. Representative image of FACS analysis with insert showing corresponding mean fluorescence intensity (n = 3).

As this was incorporating a different cell line, we initially conducted a series of preliminary experiments to ensure that both 5-FU and lithium induce autophagosome accumulation in this model. Autophagy induction in response to 5-FU, was verified by Cyto-ID autophagy detection assay, assessment of LC3 I and II by western blot, and by morphological assessment of vesicle accumulation (S6 Fig). No apoptosis was evident in this cell line. The ability of lithium to induce autophagy was also assessed. Lithium (30 mM) induced a significant increase in Cyto-ID fluorescence (2.2-fold in lithium treated compared to control), indicating autophagosome formation at 24 hours (*** p = 0.006) (Fig 4A). Morphological analysis of lithium treated cells also revealed marked accumulation of vesicles, 24 hours after treatment (Fig 4C upper right panel, arrows).

To assess if the combination of lithium and 5-FU treatment could enhance cytotoxicity in this cell model, CT26 cells were treated with 30 mM lithium and 20 μM 5-FU for 24 hours. Equal numbers of viable cells from each treatment group were seeded following drug treatment and incubated for up to two weeks, in the absence of drug. The colonies that developed were fixed, stained and are shown in Fig 4B (i). Colonies that developed in the combination treated cells were rare, with a 2.3-fold decrease in the number of colonies recovering from lithium & 5-FU treatment compared to 5-FU alone (* p = 0.0163) [Fig 4B (ii)]. Morphological analysis of combination treated cells indicated that vesicle accumulation is similar to that of cells treated with lithium alone (Fig 4C lower and upper right panels, respectively). Cyto-ID analysis of combination treated cells would again indicate that there is no greater vesicle accumulation in combination treated cells (blue overlay), when compared to single agent treated cells (red and green overlays) (Fig 4D). Collectively, these data would suggest that while both 5-FU and lithium are autophagy inducers in this cell line, the combined effect on cytotoxicity is not simply due to an additive induction of autophagy.

### Evaluation of combination treatment in a CT26 xenograft syngenic model

**Fig 5:**
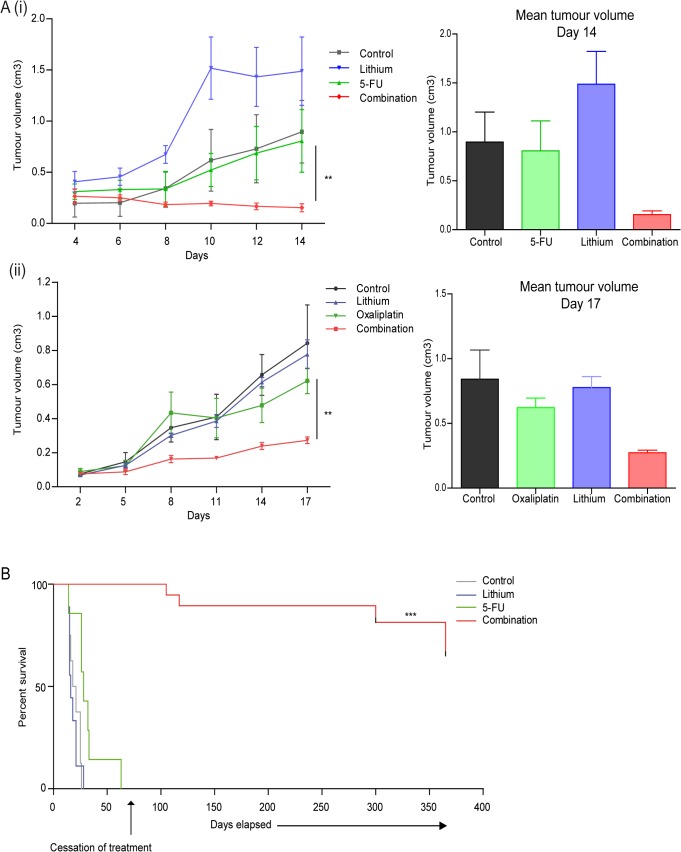
*In vivo* implementation of combination therapy in pre-clinical CT26 colorectal carcinoma model. To assess the effects of combination therapy on tumor volume and survival (A) (i) Colorectal carcinoma cells (CT26) (1 x 10^6^) were injected subcutaneously into the right flank of female balb/c mice (n = 3 per group). When mean tumor diameters were 0.5 cm +/- 0.02cm (~14 days post injection of tumor cells) all animals received an intratumoral injection every three days of either PBS (control), 5-fluorouracil (5-FU) (20 mg/kg), lithium chloride (200 mg/kg) or combinations of 5-FU and lithium chloride for two weeks. Tumor size was monitored by alternate day measurements in two dimensions, using verniers callipers, with first measurement recorded four days after initial treatment. Tumor volume was calculated according to the formula V = ab^2^ᴨ/6, where ‘a’ is the longest diameter of the tumor and ‘b’ is the longest diameter perpendicular to diameter ‘a’. (ii) CT26 cells (1 x 10^6^) were injected and tumors allowed to develop as above (n = 4 per group). To assess effects of systemically delivered combination therapy on primary tumors all treated and control groups received intraperitoneal injection every three days, of either PBS (control), oxaliplatin (10 mg/kg), lithium chloride (200 mg/kg) or combinations of oxaliplatin and lithium chloride for up to three weeks. Tumor size was assessed every three days, with the first measurement recorded two days after initial treatment. Tumor volume was calculated as above. *Asterisks* indicate a significant difference in the mean tumor volume of combination treated animals compared to single agent treated tumors (5-FU or oxaliplatin) (** p < 0.01) (unpaired *t*-test). (B) To assess the antitumor effect of combination therapy on survival, CT26 tumors were induced as above in balb/c mice (n = 8 per group) and once tumors were established all treated and control groups received an intratumoral injection every three days with PBS (control), lithium chloride (200mg/kg), 5-FU (20mg/kg) and a combination of both lithium chloride and 5-FU (200mg/kg, 20mg/kg). When tumor volume reached ~ 1.5 cm^3^ (no greater than 1.5 cm in diameter) animals were euthanized. The median survival for control treated animals was 19.5 days, lithium treated– 16 days, 5-FU treated– 28 days. Median survival of combination treated group is undefined due to long term survival of more than half the group. Treatment of the remaining combination treated animals was maintained until day 75, following which all treatments ceased. One year after treatment began these animals remained tumor free. *Asterisks* indicate a significant difference in the overall median survival of combination treated (5-FU & lithium chloride) animals compared to single agent treated animals (5-FU) (*** p < 0.0001). Statistical analysis was carried out using the log-rank test.

The promising *in vitro* effects of lithium in combination with chemotherapeutic drugs, in both the human esophageal and murine colorectal cell lines led to the examination of its anti-tumor potential *in vivo*. CT26 cells were injected subcutaneously into the right flank of adult female balb/c mice, after anesthesia. Mice were randomly divided into experimental groups and treatments were administered every three days. Tumor size was monitored by alternate day measurements and tumor volumes calculated using the formula V = ab^2^ᴨ/6, where ‘b’ is the smallest diameter, and ‘a’ the diameter measured perpendicular to ‘b’. To assess the effect of lithium (200 mg/kg), 5-fluorouracil (5-FU) (20 mg/kg) alone or in combination on CT26 tumor growth we treated tumors directly for two weeks, with the control group receiving the same volume of PBS (n = 3) [Fig 5A (i)]. While control treated tumors (black trace) or those treated with either lithium chloride or 5-FU alone (blue and green trace, respectively) increased in size over time, those treated with the combination of lithium & 5-FU (red trace) consistently reduced in size with time. At day 14, when the first of the lithium alone treated animals had to be excluded from the study, final tumor volumes were compared. A significant decrease (5.2-fold) in the mean tumor volume was observed in the combination treated animals when compared to 5-FU alone treated animals (** p = 0.007).

To assess the effect of lithium (200 mg/kg), combined with an additional chemotherapeutic—oxaliplatin (10 mg/kg) alone or in combination with lithium on tumor growth, we repeated a similar tumor growth study on CT26 derived tumors. Again, tumor size was monitored every three days, with treatment delivered every three days. Control animals received the same volume of PBS (n = 4) [Fig 5A (ii)]. While control treated tumors (black trace on graph) or those treated with either lithium chloride or oxaliplatin alone (blue and green trace, respectively) increased in size over time, those treated with the combination of lithium & oxaliplatin (red trace) developed at a much slower rate. At day 17, when the first of the control treated animals had to be excluded from the study, final tumor volumes were compared. A significant decrease (2.3-fold) in the mean tumor volume was observed in the combination treated animals when compared to oxaliplatin alone treated animals (** p = 0.004).

To assess if reducing tumor volume could influence survival in this model, we again established CT26 derived tumors and divided the animals randomly into four treatment groups, with eight animals per group. Animals were treated with either lithium chloride (200 mg/kg) or 5-FU (20 mg/kg) alone or in combination (lithium & 5-FU), with the control group receiving the same volume of PBS. In this study, animals were treated every three days and tumor volumes monitored. When tumor volume reached ~ 1.5 cm^3^ (no greater than 1.5 cm in diameter) animals were euthanized. Treatment was maintained in the combination treated group until 75 days after the beginning of the study, at which time all combination treated animals were alive and tumor free, all other groups had been euthanized. This surviving group was maintained, treatment free for a further ~ 290 days. The median survival was calculated using the log-rank test. As seen in Fig 5B median survival for control treated animals was 19.5 days (grey), lithium treated– 16 days (blue) and 5-FU treated– 28 days (green). At this point in the study median survival of the combination treated animals was undefined due to the long term survival of half of the group, beyond one year (red). When we compare the overall mean survival of the last remaining treatment group (5-FU alone) and the combination treated animals, there is a significant increase in survival of the combination treated group (*** p < 0.0001).

Collectively these data demonstrate that combining lithium and a chemotherapeutic can significantly reduce CT26 tumor burden in this murine model, when compared to treatment with either lithium, 5-FU or oxaliplatin alone. This combination treatment can also dramatically increase the survival of animals, well beyond that of single agent treated animals.

## Discussion

Most therapeutic strategies aimed at autophagy modulation in cancer are targeting autophagy inhibition due to well-characterised effects of autophagy on cell survival. In this study, we have examined the less conventional approach of testing established autophagy inducers in cancer cells. It is clear from our data that autophagy inducers cannot be considered as a singular group, as their mechanism of action is critical. An mTOR inhibitor could not chemosensitize apoptosis incompetent esophageal cancer cells, whereas lithium could. The effects of these inhibitors can of course always be attributed to other ‘non-autophagic’ activity. This is true of most available autophagy modulators. However, as we study modulators that are capable of chemosensitizing, it is hoped that we may identify common mechanisms of action that will assist the future development of more specific compounds. This current study suggests that lithium is an autophagy modulator that may also compromise lysosome integrity, leading to an added vulnerability when treated with a chemotherapeutic drug.

In our analysis of esophageal cells, rapamycin was found to induce autophagy but could not enhance chemosensitivity. Indeed, it was slightly protective. This is consistent with known survival functions of autophagy, but would not be supportive of clinical use. Derivatives of rapamycin (rapalogues)—everolimus, temsirolimus have been FDA approved for the treatment of advanced/metastatic renal cell carcinoma. Preclinical studies with rapamycin had indicated both cytostatic activity and the ability of rapamycin to sensitize cells to apoptosis [32].

However, despite initially promising preclinical and clinical data in renal cell carcinoma, studies now suggest that as single agents, the activity of rapalogues is modest and sporadic in other solid tumors. A number of reasons for this limited success have been proposed. Firstly, rapamycin inhibits only mTORC1 and in doing so it also inhibits a strong mTORC1-dependent negative feedback loop, leading to activation of Akt and increased tumor cell survival [33]. Its weak activity against mTORC2 (Akt activator) may also limit its effectiveness. Other second generation mTOR inhibitors and dual mTORC1 and 2 inhibitors are under evaluation, in addition to novel drugs that block co-operating signaling molecules such as PI3K, Akt and PDK-1. Some have shown promise in preclinical and early clinical studies [32] [34]. The ultimate success of these agents will require a better understanding of the determinants of response.

It has been reported that synergy between rapamycin and carboplatin or paclitaxel, requires sequential treatment of cells with chemotherapy followed by rapamycin, to optimize induction of apoptosis [35]. In addition, other studies have reported that rapamycin or its derivatives only induce cell death if the cells are PI3K/Akt/mTOR addicted and apoptosis competent [36]. This would be consistent with a lack of chemosensitization in our apoptosis incompetent cell lines (KYSE450 and OE19) despite clear evidence of autophagy induction (and mTOR inhibition; dephosphorylation of p70S6K and mTOR, data not shown). In contrast, chemosensitization (promotion of apoptosis) is achieved in apoptosis competent (OE21 and OE33) cells. A companion biomarker of apoptotic response would therefore be of major value in trials with mTOR inhibitors, as these drugs may only be effective in apoptosis competent cancers.

Lithium salts have been previously reported to have anti-neoplastic activity due to inhibition of GSK-3β signaling [37–40]. Enhanced GSK-3β expression in colon carcinoma samples was associated with resistance to 5-FU and *in vitro* inhibition of GSK-3β abolished cell viability via necroptosis and reduced xenograft growth *in vivo* [41]. More recently, lithium has been shown to synergistically enhance the anticancer effects of gemcitabine in pancreatic ductal adenocarcinoma cells [42], ionizing radiation in breast cancer cells [43] and sorafenib in glioblastoma multiforme cells [44] through targeting of GSK-3β signaling and induction of apoptotic cell death. Other studies have also indicated that alone, lithium has cytotoxic effects via TNF-α and FasL-mediated apoptosis [45, 46]. Although lithium has been reported to enhance cytotoxicity of anticancer agents, these studies consistently link the effects of lithium to apoptosis. Indeed, we also found that lithium promoted apoptosis in the apoptosis competent esophageal cell lines, OE21 and OE33. However, our study also reports lithium-induced autophagy and a chemosensitizing activity in apoptosis incompetent cells, leading to the induction of a non-apoptotic/Type II-like cell death. This could not be mimicked by GSK inhibition (data not shown) suggesting that other lithium targets are involved. To our knowledge, this has not been previously reported in cancer.

The nature of the cell death induced by the combination of lithium and 5-FU is currently undefined. It exhibits features of Type II, but we cannot fulfil the current criteria required to refer to it as autophagic cell death [47]. Other studies have reported autophagy mediated cell death in cancer cells following treatment with chemotherapeutic agents [3], particularly in apoptosis defective cells [2]. Apoptosis-resistant glioma cells were sensitized to temozolomide via autophagic cell death [48] and p53 has been linked to enhanced autophagic cell death in response to an anticancer agent in colon cancer cells [49]. Autophagic cell death regulated by caspase 10 has also been reported in multiple myeloma [50] and acute Ras overexpression can lead to autophagic cell death [47]. Developmental studies in lower animals also indicate that a regulated, programmed form of autophagic cell death exists [51, 52]. In addition, a morphologically distinct and regulated form of autophagic cell death has recently been described in mammalian cells and referred to as autosis. This can be triggered by an autophagy inducing peptide (TAT-Beclin 1) or starvation and is dependent upon the Na^+^, K^+^-ATPase [53]. It has also been proposed that the outcome of autophagy, in terms of survival or death is solely related to a cells capacity to produce sufficient ATP during a stress response. Adequate metabolic fitness was suggested to determine whether a cell survives or dies by apoptosis or necrosis [54]. It is possible that defective lysosome function in lithium treated cells may lead to depletion of key metabolites and this could negatively impact on ‘metabolic fitness’ and viability.

Other studies of autophagy in cardiomyocytes following ischemia-reperfusion injury have demonstrated that compromised flux can influence viability. BNIP3 expression induced autophagosome accumulation with lysosome consumption and increased susceptibility to cell death. Forced expression of TFEB, a transcriptional regulator of lysosome biogenesis restored autophagosome processing and protected against cell death [27]. Another study of ischemia-reperfusion injury found autophagosome accumulation and depletion of LAMP2. Restoration of LAMP2 restored autophagosome processing and protected cardiomyocytes from cell death [26]. Impairment of autophagic flux by B10, a derivative of betulinic acid was also reported to convert autophagy in glioblastoma cells into a cell death process associated with lysosomal destabilization [55] and the anti-tumour activity of recombinant Galectin 9 was also associated with impairment of lysosomal function and fatal frustration of autophagy [56]. However, our data would suggest that a blockage in flux alone is insufficient to promote cytotoxicity, as lysosomal inhibitors do not convert rapamycin into a cytotoxic agent in these cells. It is possible that the defects we observed in lysosomal proteins–in addition to flux–contribute to cell death. Lysosomal mediated cell death has been previously described with both apoptotic and several non-apoptotic pathways reported [30]. This type of cell death is associated with lysosomal membrane permeabilization (LMP) and release of lysosomal hydrolases. The causes of LMP include deficiencies in LAMP 1/2 proteins, increased cytosolic calcium, imbalance in specific membrane lipids (sphingolipids & cholesterol) and reactive oxygen species [57]. Further investigation would be required to define the contribution of autophagy and lysosomes to chemosensitization with lithium. It remains possible that the effects on autophagy and lysosomes may not be responsible for the chemosensitizing activity of lithium. Lithium may have other independent effects that compromise cell viability.

Our preliminary data with lithium would encourage further evaluation in other models and indeed investigation of other autophagy modulators. The concept of using a known ‘autophagy inducer’ in cancer will however appear inconsistent with current clinical trials with autophagy inhibitors. More than 30 phase I/II cancer clinical trials are underway to assess the benefits of chloroquine or its derivative hydroxychloroquine (FDA approved autophagy inhibitors) in combination with conventional chemotherapy [2, 58]. However, mechanistically, chloroquine is not an autophagy inhibitor–but a blocker of autophagic flux. Our *in vitro* data did not support the use of chloroquine as a chemo-sensitiser in esophageal cancer cells, whereas we did report chemo-sensitisation in leukaemia cells [59]. Further mechanistic work is therefore required to establish the mechanisms involved.

A number of recent studies support an autophagy induction approach for cancer. Lisanti *et al*. [60] have described an important balance between autophagy in cancer associated fibroblasts (CAFs) and tumor cells. These CAFs exhibit heightened autophagy and mitophagy, leading to nonaerobic glycolysis and the ‘Reverse Warbug effect’ [60]. This enables the stromal cells to ‘feed’ high-energy metabolites (lactate/glutamine/ketone bodies/fatty acids) to the cancer cells, which downregulate autophagy. This model predicts that either an autophagy inducer or an autophagy inhibitor would disrupt this balance through actions on the tumor cells or CAFs respectively and therefore, both strategies are therapeutically relevant [61, 62]. In addition, several studies have now demonstrated that activation of immune cells is critical for the success of chemotherapeutic regimes. Chemotherapeutic agents induce the release of damage-associated molecular patterns (DAMPs) which prime effector immune cells to induce immunogenic cancer cell death [63]. Importantly, a recent study has shown that effective induction of autophagy in tumor cells by chemotherapy is a critical requirement for the establishment of an antitumor immune response [64, 65]. It will be difficult to establish whether chloroquine will impede immunogenic cell death in the current clinical trials. Limited studies have been done in *in vivo* models, with most performed in immune-compromised animals [66]. In our animal studies, all of the above mechanisms could potentially have contributed to the success of the regime and further mechanistic studies are warranted.

It is now apparent that autophagy can influence several aspects of cancer biology. The challenge now is how best to manipulate it for better therapeutic gain. Lithium, as a mood stabilizer, has a long history of safety in the clinic and indeed recommendation of more widespread use was suggested following a recent meta-analysis [67–69]. Side effects are manageable and this agent shows excellent promise for new clinical trials to assess chemosensitization. In our animal studies, no lithium toxicity was evident and a remarkable increase in tumor free survival was achieved with the combination treatment. This represents an exciting new avenue for therapeutic intervention, particularly for apoptosis incompetent cancers.

## Supporting Information

S1 Fig Assessment of autophagic flux in KYSE450 esophageal cells. **A** Cells were pretreated with chloroquine (10 μM) for two hours, prior to treatment with 5-fluorouracil (5-FU) (30 μM) for 48 hours and autophagy levels assessed with the Cyto-ID autophagy detection kit. Representative image of FACS analysis (**i**), with data presented to the right as mean fluorescence intensity (ii). *Asterisks* indicate a significant difference in mean fluorescence intensity in combination treated cells compared to single agent treated cells (*** p < 0.001, ** p < 0.01) (unpaired *t*-test).
(TIF)

S2 Fig Lithium enhanced sensitivity of OE19 and KYSE450 (drug resistant apoptotic incompetent) cells to chemotherapeutics. Both OE19 and KYSE450 cell lines were seeded and treated with combinations of lithium (30 mM) without or with 5-FU (40 μM) or cisplatin (20 μM) for 48 hours. Following treatment, viable cells were counted and equal numbers reseeded in triplicate, in the absence of drug. Cells were allowed to adhere and grow for 14 days, they were then fixed and stained and regrowth of colonies examined. Data demonstrates that both drug resistant esophageal cancer cell lines are sensitized to both chemotherapeutic agents by the addition of lithium.
(TIF)

S3 Fig Induction of autophagy with lithium or rapamycin enhanced apoptotic cell death in OE21 esophageal cells. OE21 esophageal cancer cells were treated with lithium chloride (30 mM) or rapamycin (300 nM), without and with 5-fluorouracil (5-FU) (30 μM) for 48 hours. Morphological features of the OE21 cells treated with lithium **A (i)** or rapamycin **B (i)** alone or in combination with 5-FU is shown and apoptosis is indicated with arrows (Magnification 40x). An MTT assay was used to assess the effects of combination treatment on the viability of cells treated with lithium **A (ii)** or rapamycin **B (ii)** in combination with 5-FU. These data are presented as mean +/- SEM of three independent experiments. Data demonstrates that autophagy inducers, in apoptotic competent cells, induce both apoptosis and autophagy and enhance sensitivity of these cells to the chemotherapeutic drug 5-FU.
(TIF)

S4 Fig Confirms lithium specific effects are common to other lithium salts. KYSE450 cells were treated with a range of concentrations of lithium carbonate (Li_2_CO_3_) (10–20 mM) alone and in combination with 5-fluorouracil (5-FU) (30 μM) for 48 hours. **A** Western blot analysis of LC3 expression in cells treated with lithium carbonate (10, 12 and 15 mM) for 48 hours shows a clear elevation of LC3 I and LC3 II at all concentrations examined. β-actin is used as a loading control. **B** Immunofluorescent staining of LC3 in lithium (carbonate and chloride) treated cells demonstrates LC3 positive stained cells with both lithium salts. **C** Morphological features of KYSE450 cells were examined following 48 hours treatment. Arrows indicate accumulation of cytoplasmic vesicles in a pattern similar to that observed with lithium chloride, while CD denotes morphological changes associated with cell death (Magnification 40x). **D** A colony formation assay was used to assess if lithium carbonate sensitized cells to 5-FU. Viable cells following 48 hour treatment with 5-FU in the absence or presence of lithium carbonate were counted and equal numbers reseeded in triplicate, in the absence of drug. Cells were allowed to grow for 14 days, they were then fixed and stained and colonies quantified using the Odyssey Infrared Imaging System (Li-COR). Data is presented graphically as the mean +/- SEM of three independent experiments (*** p < 0.0005, ** p < 0.005). Data indicates that lithium carbonate is a powerful inducer of autophagy and a chemosensitizer in esophageal cancer cells.
(TIF)

S5 Fig Demonstrates that inhibition of rapamycin induced autophagic flux with lysosomal inhibitors—bafilomycin or chloroquine, in KYSE450 esophageal cells is not cytotoxic or chemosensitizing. KYSE450 cells treated with rapamycin (200 & 300 nM), bafilomycin (1 nM), chloroquine (10 μM) and 5-fluorouracil (5-FU) (30 μM) for 24 and 48 hours. **A (i)** Western blot analysis of LC3 expression shows a clear inhibition of autophagic flux with bafilomycin. All bands were quantified using the Odyssey Imaging System, normalized to β-actin and presented as integrated intensities to the right. **(ii)** Following 48 hours of treatment, viable cells were counted and equal numbers reseeded in triplicate, in the absence of drug. Cells were allowed to adhere and grow for 14 days, fixed and stained and regrowth of colonies quantified, data shown to the right. No enhancement of cytotoxicity was observed. **B (i)** Cells were pretreated with chloroquine (10 μM) for two hours, prior to treatment with rapamycin (300 nM) for 24 hours and autophagy levels assessed with the Cyto-ID autophagy detection kit. Representative image of FACS analysis, with data presented to the right as mean fluorescence intensity. *Asterisks* indicate a significant difference in mean fluorescence intensity in combination treated cells compared to single agent treated cells (*** p < 0.001) (unpaired *t*-test). **(ii)** Cells were treated with either rapamycin (300 nM), chloroquine (10 μM) or a combination of both for 48 hours. Colony regrowth was assessed as above, with data presented graphically to the right. Collectively these data suggest that an impediment in flux is not necessarily a cytotoxic or chemosensitizing event.
(TIF)

S6 Fig Examines the effects of 5-fluorouracil (5-FU) on autophagy in the CT26 murine colorectal cell line. CT26 cells were treated with either 5-FU (10–30 μM) alone or in combination with chloroquine (10 μM) for 24 hours. **A** Cells were assessed for autophagy induction 24 hours after treatment with 5-FU, with the Cyto-ID autophagy detection kit. Panel on the left show representative image of FACS analysis, with panel to the right showing corresponding mean fluorescence intensity. Increased Cyto-ID fluorescence indicated significant autophagosome formation in 5-FU (10, 20 and 30 μM) treated cells, at 24 hours (> fourfold increase observed between control and 5-FU treated cells). **B** Western blot analysis of LC3 expression in cells treated with 5-FU for 24 hours confirmed an increase in the levels of LC3 II. LC3I and LC3II bands were quantified using the Odyssey Infrared Imaging System (Li-COR), normalized to β-actin and presented as integrated intensities. **C** Morphological analysis of CT26 cells confirmed accumulation of vesicles in the cytoplasm of cells treated with 5-FU (10–30 μM) for 24 hours. Black arrows indicate accumulation of vesicles (Magnification 40x).
(TIF)

S7 Fig Un-cropped western blots. (TIF)

S1 File ARRIVE Guidelines. (PDF)
