# Supplementary figures and images for: Lithium Modulates Autophagy in Esophageal and Colorectal Cancer Cells and Enhances the Efficacy of Therapeutic Agents In Vitro and In Vivo
Source: PLoS One. 2015 Aug 6;10(8):e0134676. doi: 10.1371/journal.pone.0134676 (PMC4527721; doi:10.1371/journal.pone.0134676)

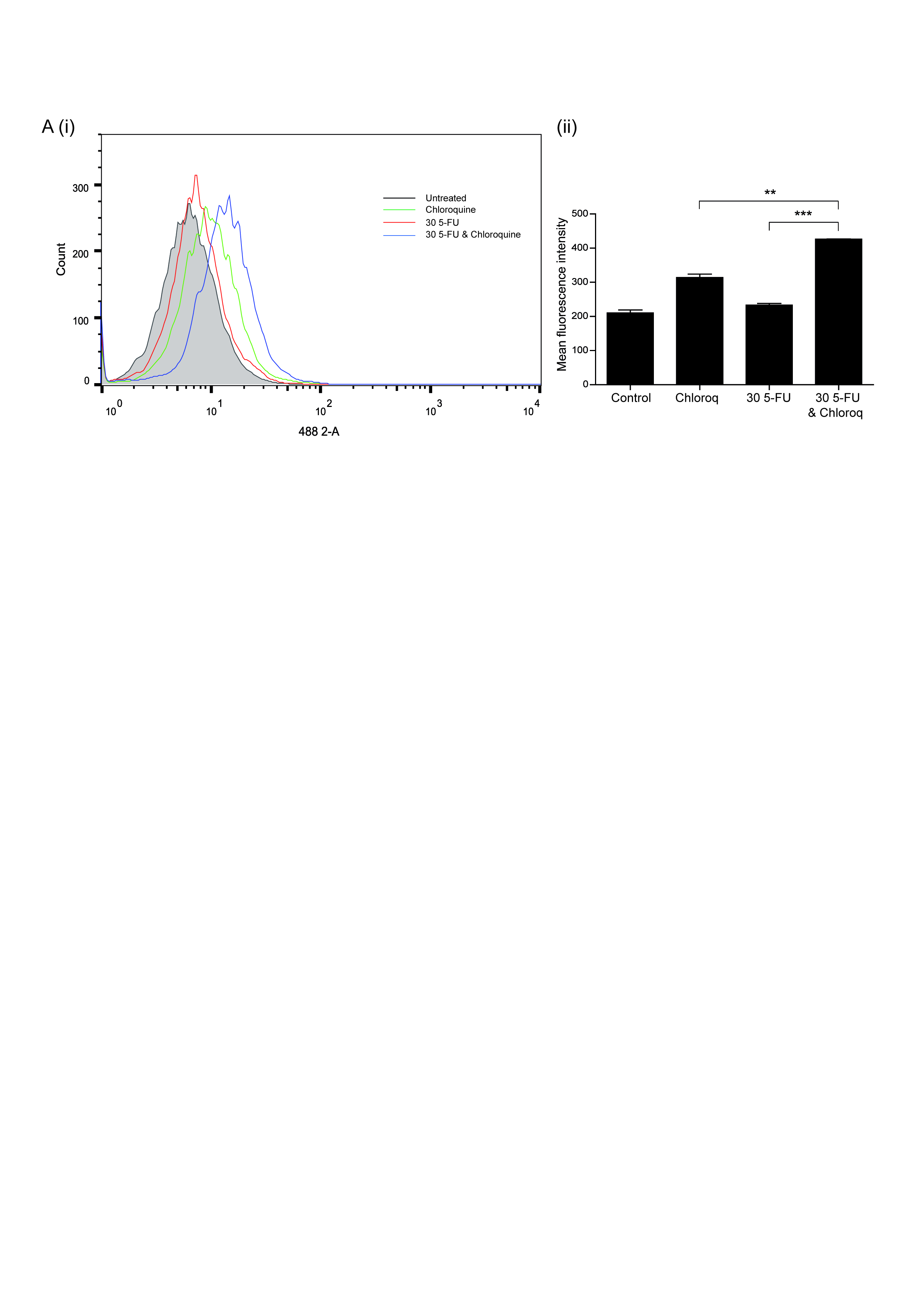

Supplement: S1 Fig — A Cells were pretreated with chloroquine (10 μM) for two hours, prior to treatment with 5-fluorouracil (5-FU) (30 μM) for 48 hours and autophagy levels assessed with the Cyto-ID autophagy detection kit. Representative image of FACS analysis (i), with data presented to the right as mean fluorescence intensity (ii). Asterisks indicate a significant difference in mean fluorescence intensity in combination treated cells compared to single agent treated cells (*** p < 0.001, ** p < 0.01) (unpaired t-test). (TIF) [file pone.0134676.s001.tif]

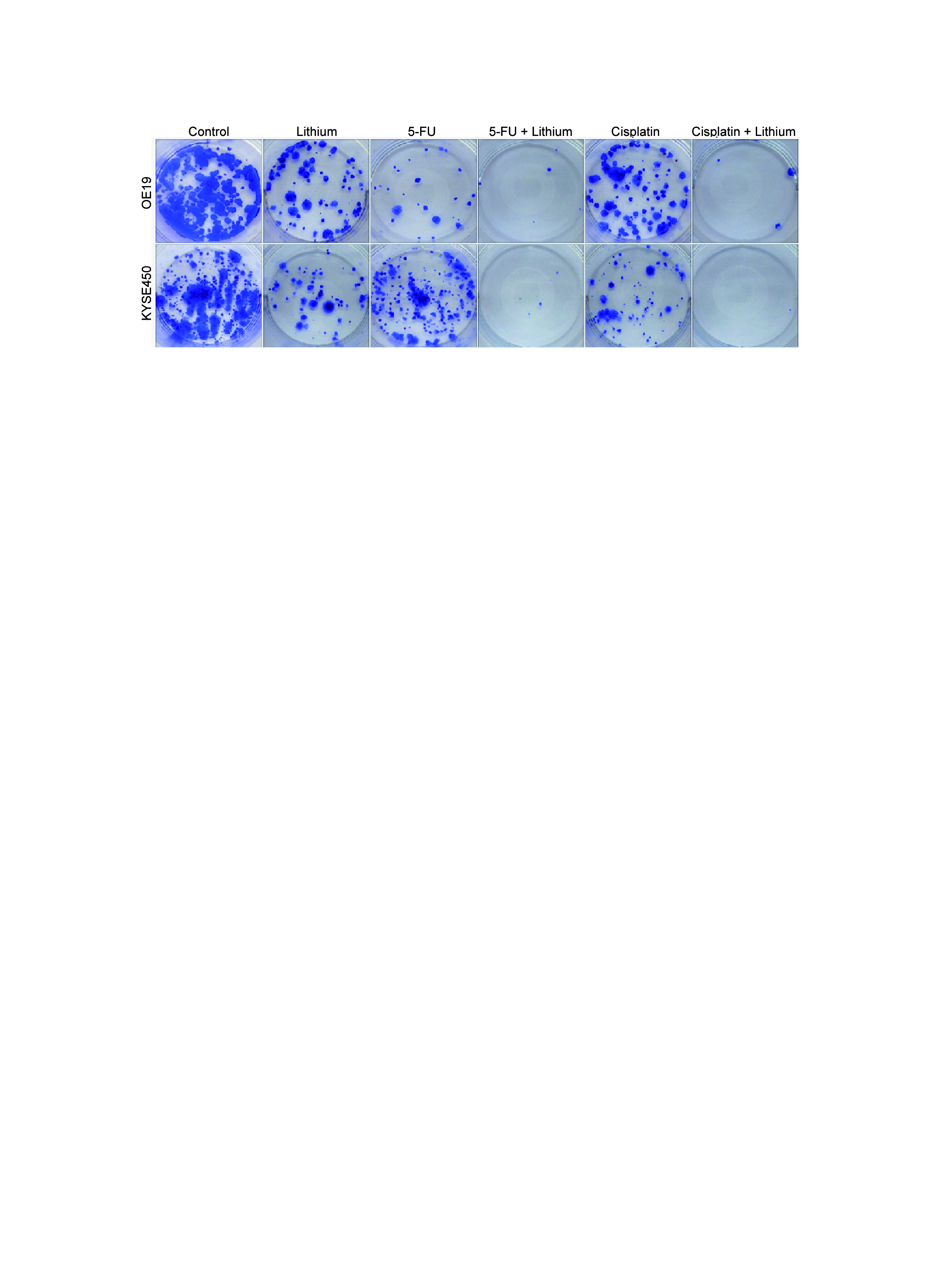

Supplement: S2 Fig — Both OE19 and KYSE450 cell lines were seeded and treated with combinations of lithium (30 mM) without or with 5-FU (40 μM) or cisplatin (20 μM) for 48 hours. Following treatment, viable cells were counted and equal numbers reseeded in triplicate, in the absence of drug. Cells were allowed to adhere and grow for 14 days, they were then fixed and stained and regrowth of colonies examined. Data demonstrates that both drug resistant esophageal cancer cell lines are sensitized to both chemotherapeutic agents by the addition of lithium. (TIF) [file pone.0134676.s002.tif]

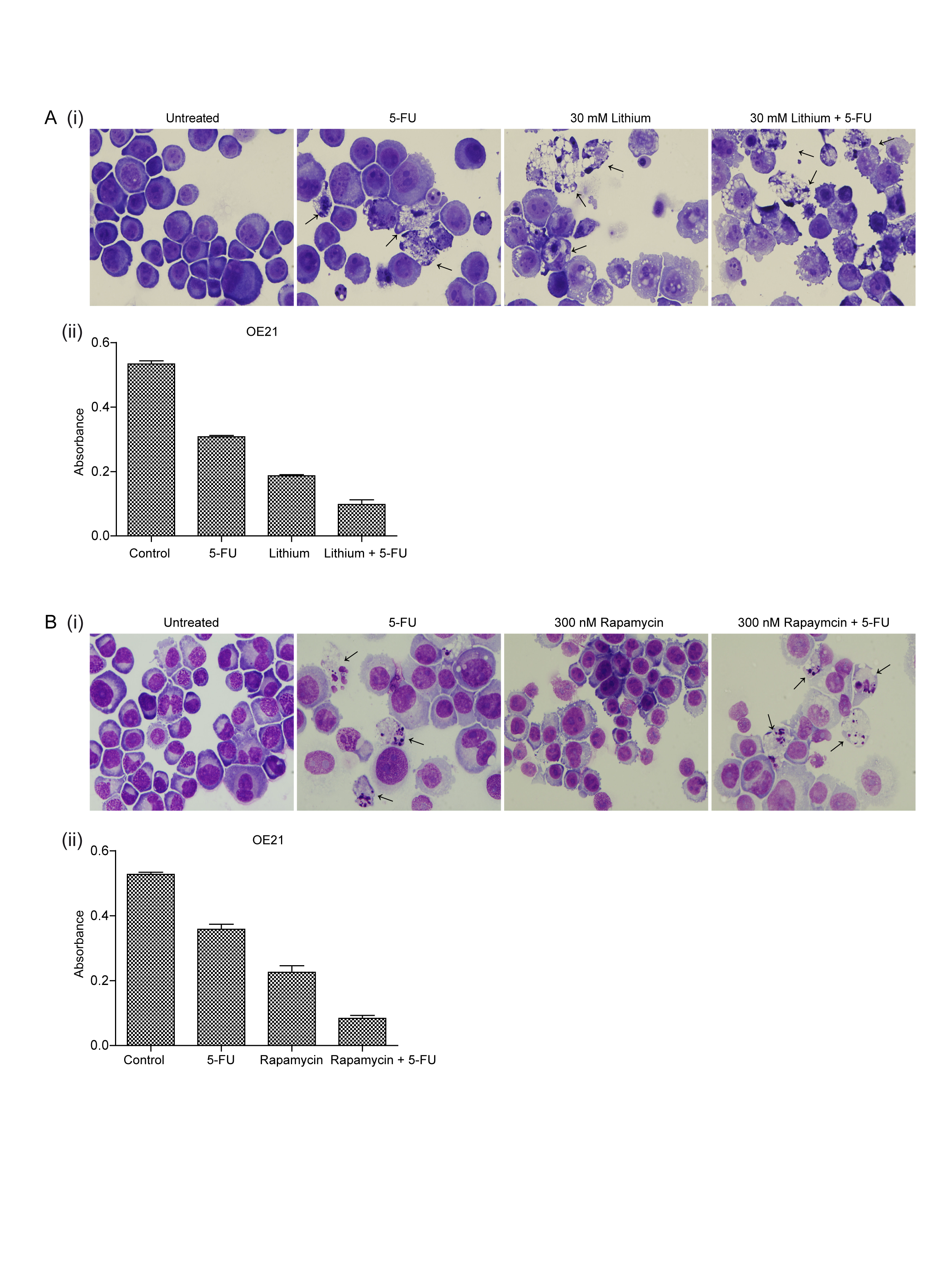

Supplement: S3 Fig — OE21 esophageal cancer cells were treated with lithium chloride (30 mM) or rapamycin (300 nM), without and with 5-fluorouracil (5-FU) (30 μM) for 48 hours. Morphological features of the OE21 cells treated with lithium A (i) or rapamycin B (i) alone or in combination with 5-FU is shown and apoptosis is indicated with arrows (Magnification 40x). An MTT assay was used to assess the effects of combination treatment on the viability of cells treated with lithium A (ii) or rapamycin B (ii) in combination with 5-FU. These data are presented as mean +/- SEM of three independent experiments. Data demonstrates that autophagy inducers, in apoptotic competent cells, induce both apoptosis and autophagy and enhance sensitivity of these cells to the chemotherapeutic drug 5-FU. (TIF) [file pone.0134676.s003.tif]

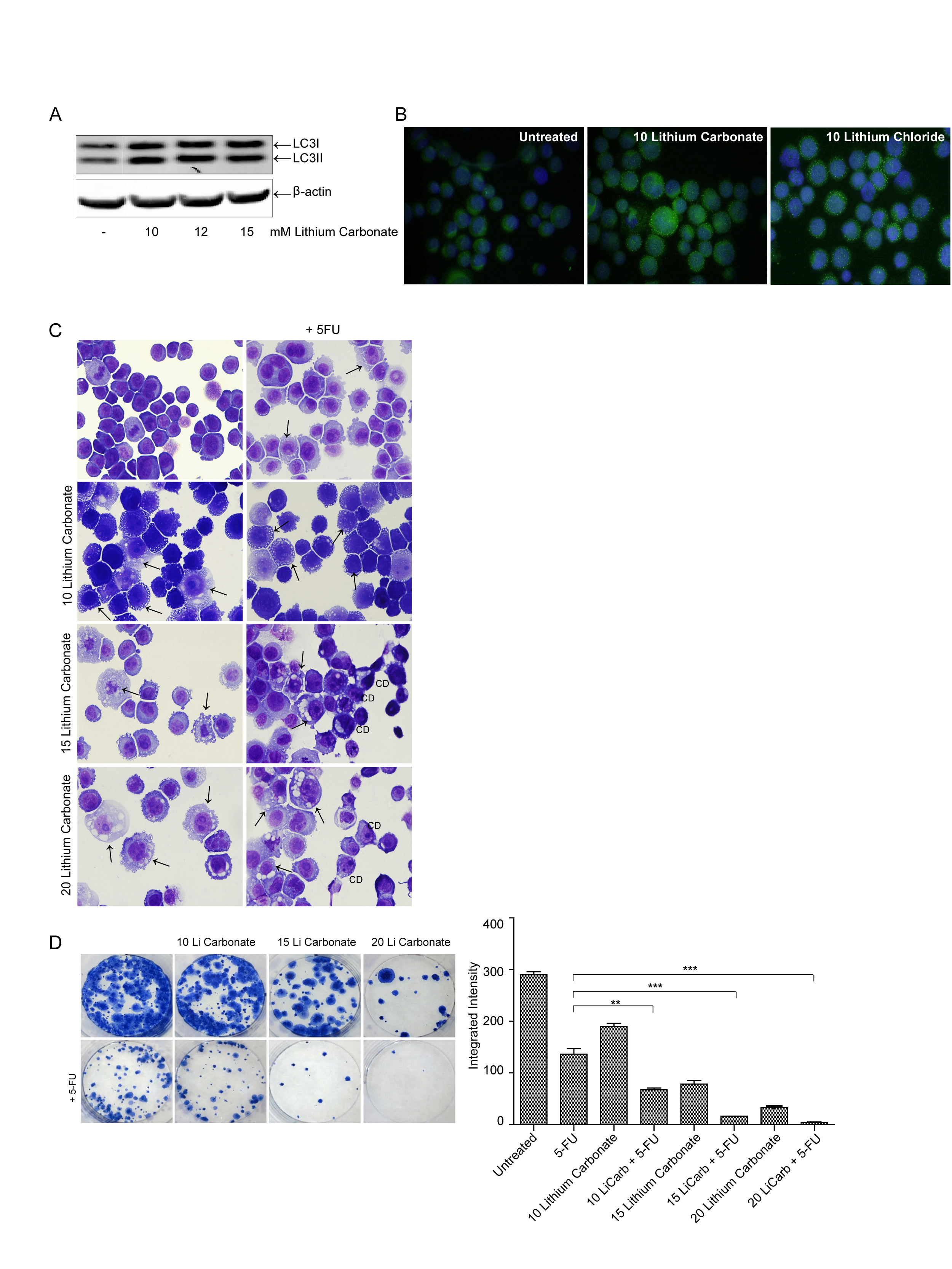

Supplement: S4 Fig — KYSE450 cells were treated with a range of concentrations of lithium carbonate (Li2CO3) (10–20 mM) alone and in combination with 5-fluorouracil (5-FU) (30 μM) for 48 hours. A Western blot analysis of LC3 expression in cells treated with lithium carbonate (10, 12 and 15 mM) for 48 hours shows a clear elevation of LC3 I and LC3 II at all concentrations examined. β-actin is used as a loading control. B Immunofluorescent staining of LC3 in lithium (carbonate and chloride) treated cells demonstrates LC3 positive stained cells with both lithium salts. C Morphological features of KYSE450 cells were examined following 48 hours treatment. Arrows indicate accumulation of cytoplasmic vesicles in a pattern similar to that observed with lithium chloride, while CD denotes morphological changes associated with cell death (Magnification 40x). D A colony formation assay was used to assess if lithium carbonate sensitized cells to 5-FU. Viable cells following 48 hour treatment with 5-FU in the absence or presence of lithium carbonate were counted and equal numbers reseeded in triplicate, in the absence of drug. Cells were allowed to grow for 14 days, they were then fixed and stained and colonies quantified using the Odyssey Infrared Imaging System (Li-COR). Data is presented graphically as the mean +/- SEM of three independent experiments (*** p < 0.0005, ** p < 0.005). Data indicates that lithium carbonate is a powerful inducer of autophagy and a chemosensitizer in esophageal cancer cells. (TIF) [file pone.0134676.s004.tif]

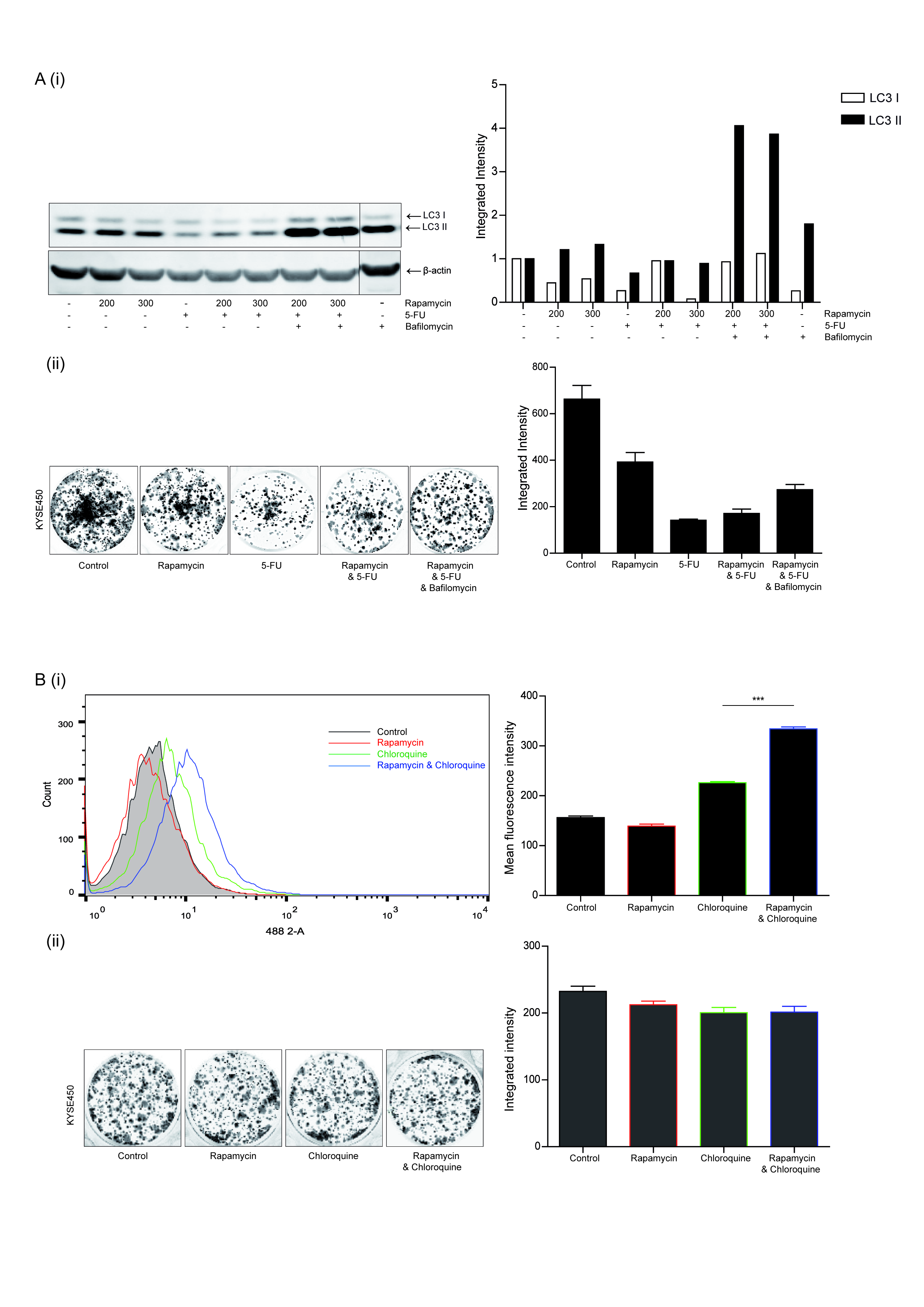

Supplement: S5 Fig — KYSE450 cells treated with rapamycin (200 & 300 nM), bafilomycin (1 nM), chloroquine (10 μM) and 5-fluorouracil (5-FU) (30 μM) for 24 and 48 hours. A (i) Western blot analysis of LC3 expression shows a clear inhibition of autophagic flux with bafilomycin. All bands were quantified using the Odyssey Imaging System, normalized to β-actin and presented as integrated intensities to the right. (ii) Following 48 hours of treatment, viable cells were counted and equal numbers reseeded in triplicate, in the absence of drug. Cells were allowed to adhere and grow for 14 days, fixed and stained and regrowth of colonies quantified, data shown to the right. No enhancement of cytotoxicity was observed. B (i) Cells were pretreated with chloroquine (10 μM) for two hours, prior to treatment with rapamycin (300 nM) for 24 hours and autophagy levels assessed with the Cyto-ID autophagy detection kit. Representative image of FACS analysis, with data presented to the right as mean fluorescence intensity. Asterisks indicate a significant difference in mean fluorescence intensity in combination treated cells compared to single agent treated cells (*** p < 0.001) (unpaired t-test). (ii) Cells were treated with either rapamycin (300 nM), chloroquine (10 μM) or a combination of both for 48 hours. Colony regrowth was assessed as above, with data presented graphically to the right. Collectively these data suggest that an impediment in flux is not necessarily a cytotoxic or chemosensitizing event. (TIF) [file pone.0134676.s005.tif]

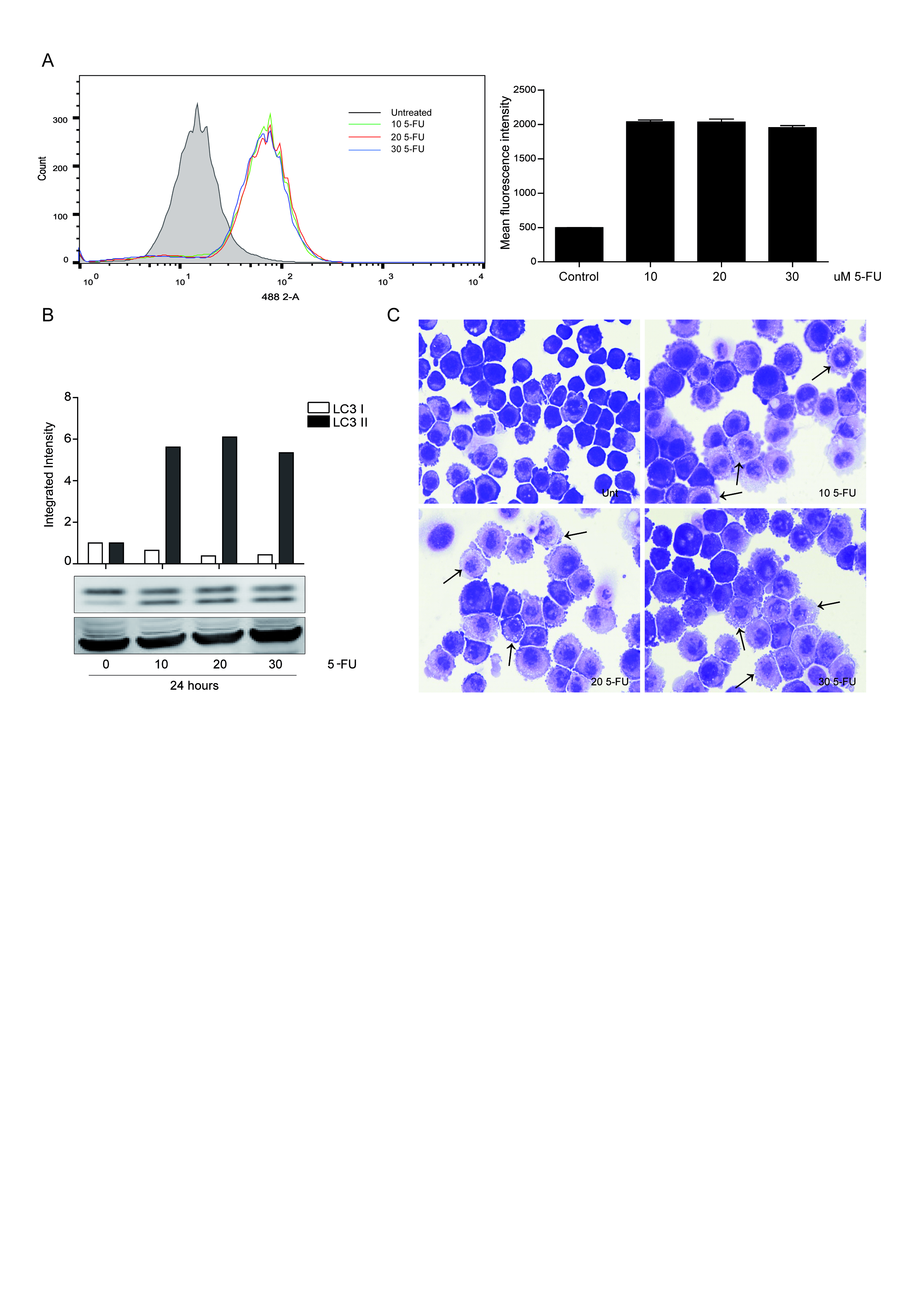

Supplement: S6 Fig — CT26 cells were treated with either 5-FU (10–30 μM) alone or in combination with chloroquine (10 μM) for 24 hours. A Cells were assessed for autophagy induction 24 hours after treatment with 5-FU, with the Cyto-ID autophagy detection kit. Panel on the left show representative image of FACS analysis, with panel to the right showing corresponding mean fluorescence intensity. Increased Cyto-ID fluorescence indicated significant autophagosome formation in 5-FU (10, 20 and 30 μM) treated cells, at 24 hours (> fourfold increase observed between control and 5-FU treated cells). B Western blot analysis of LC3 expression in cells treated with 5-FU for 24 hours confirmed an increase in the levels of LC3 II. LC3I and LC3II bands were quantified using the Odyssey Infrared Imaging System (Li-COR), normalized to β-actin and presented as integrated intensities. C Morphological analysis of CT26 cells confirmed accumulation of vesicles in the cytoplasm of cells treated with 5-FU (10–30 μM) for 24 hours. Black arrows indicate accumulation of vesicles (Magnification 40x). (TIF) [file pone.0134676.s006.tif]

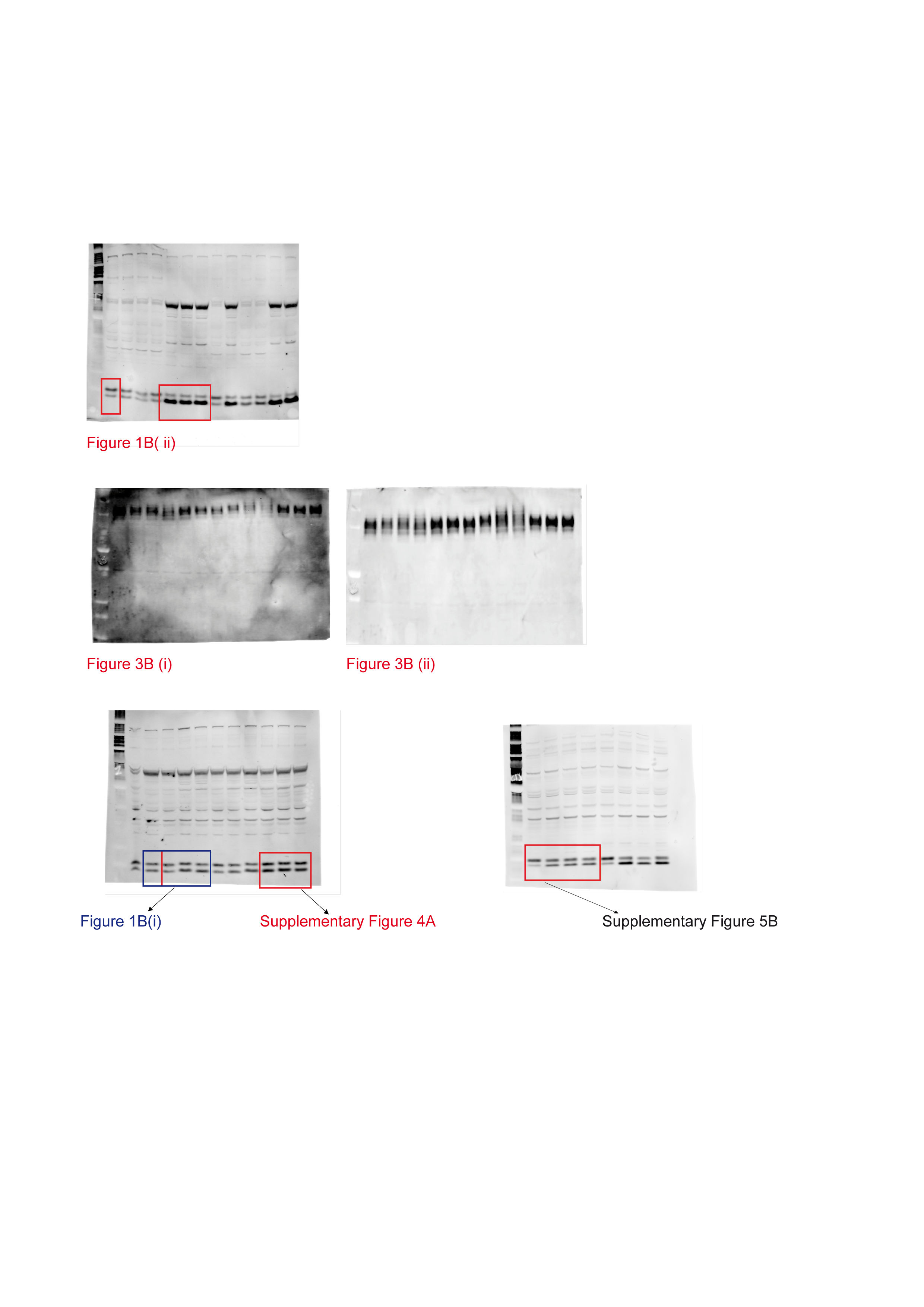

Supplement: S7 Fig — (TIF) [file pone.0134676.s007.tif]
